# Derepression of the USP22-FASN axis by p53 loss under oxidative stress drives lipogenesis and tumorigenesis

**DOI:** 10.1038/s41420-022-01241-9

**Published:** 2022-11-04

**Authors:** Zelong Han, Ming Liu, Yuxin Xie, Kunlin Zeng, Ziling Zhan, Yanwen Chen, Li Wang, Xiaoxia Chen, Yaxin Luo, Yu Zeng, Hongchao Zhan, Yingzhuo Lin, Keqin Zhang, Xiaoxia Zhu, Side Liu, Xiaobei Luo, Aidong Zhou

**Affiliations:** 1 Department of Cell Biology, School of Basic Medical Science, Southern Medical University, Guangzhou, 510515 China; 2 Guangdong Provincial Key Laboratory of Gastroenterology, Department of Gastroenterology, Nanfang Hospital, Southern Medical University, Guangzhou, 510515 China; 3 Department of Gastroenterology, Yangjiang People’s Hospital, Yangjiang, 529599 China; 4 Department of Oncology, The Fifth Affiliated Hospital, Southern Medical University, Guangzhou, 510999 China; 5 Department of Radiation Oncology, Zhujiang Hospital, Southern Medical University, Guangzhou, 510285 China; 6 Guangdong Province Key Laboratory of Molecular Tumor Pathology, School of Basic Medical Science, Southern Medical University, Guangzhou, 510515 China

**Keywords:** Stress signalling, Oncogenes, Ubiquitylation

## Abstract

Overproduction of reactive oxygen species (ROS) and aberrant lipid metabolism are established hallmarks of cancer; however, the role of ROS in lipid synthesis during tumorigenesis is almost unknown. Herein, we show that ROS regulates lipid synthesis and thus controls colorectal tumorigenesis through a p53-dependent mechanism. In p53 wild-type colorectal cancer (CRC) cells, hydrogen peroxide (H_2_O_2_)-induced p53 expression represses the transcription of deubiquitinase USP22, which otherwise deubiquitinates and stabilizes Fatty Acid Synthase (FASN), and thus inhibits fatty acid synthesis. Whereas, in p53-deficient CRC cells, ROS-mediated inhibition of USP22 is relieved, leading to FASN stabilization, which thus promotes lipid synthesis and tumor growth. In human CRC specimens, USP22 expression is positively correlated with FASN expression. Our study demonstrates that ROS critically regulates lipid synthesis and tumorigenesis through the USP22-FASN axis in a p53-dependent manner, and targeting the USP22-FASN axis may represent a potential strategy for the treatment of colorectal cancer.

## Introduction

Oxidative stress (OS), defined as a relative excess of reactive oxygen species (ROS), is one of the hallmarks of human cancer. Overproduction of ROS in tumor is usually caused by increased metabolic rate, genetic aberrations, and relative hypoxia [1]. ROS has a dual role in cancer. On the one hand, ROS can promote pro-tumorigenic signaling, facilitating cancer cell proliferation and tumor growth [1, 2]. On the other hand, ROS promotes anti-tumorigenic signaling and triggers oxidative stress-induced cancer cell death [1, 3]. However, the underlying mechanism that determines the pro-survival or pro-apoptotic effect of ROS remains largely elusive.

Loss-of-function (LOF) of p53 is a critical pro-tumorigenic event associated with abnormalities of genome integrity, cell cycle, and redox homeostasis [4]. Genetic alteration of p53 has been found in over 60% of colorectal cancers (CRCs) [5]. p53 is induced and activated by ROS, and mediates an antioxidant response to prevent the activation of oxidative stress-activated signaling pathways, such as PI3K-Akt and mTOR [6–8]. p53 mutation fails to exert antioxidant activities and maintain genome integrity, but rather increases ROS unbalance and thus promotes pro-tumorigenic pathways [9]. However, the functional mechanism of p53 in mediating ROS-induced cellular responses requires further elucidation.

Cancer cells rely on fatty acids (FAs) synthesis to convert nutrients into metabolic intermediates for membrane biosynthesis, energy storage, and the generation of signaling molecules [10]. Fatty acid synthase (FASN) is a multi-enzyme protein that catalyzes the synthesis of FAs from acetyl-CoA and malonyl-CoA in the presence of nicotinamide adenine dinucleotide phosphate (NADPH) [11]. The function of FASN in cancer has been intensively studied, and targeting FASN represents a promising therapeutic strategy for multiple cancers [12, 13]. Moreover, it is believed that wild-type p53 enhances fatty acid oxidation while inhibiting FAs synthesis in both normal and tumor cells [14], but the underlying mechanism is not well understood.

A few studies have investigated the role of ROS in lipid synthesis in different biological contexts, but have yielded inconsistent results. In neurons, the elevation of ROS from defective mitochondrial promotes FA synthesis and accumulation of lipid droplets, leading to neurodegeneration [15]. Moreover, ROS is overproduced with aging and induces acetyl-CoA production and lipid accumulation in the liver [16]. On the contrary, oxidative stress can attenuate lipid synthesis and promote fatty acid oxidation in hepatoma cells [17]. Similar results have also been demonstrated in astrocytes where overproduced ROS decreases lipid droplet accumulation and thus leads to elevated ROS toxicity [18]. However, until recently, the effect of overproduced ROS on lipid synthesis in tumorigenesis is almost unknown. In this study, we have found that H_2_O_2_ regulates FASN ubiquitination, stability, and lipid synthesis in CRC cells through a p53-dependent mechanism. We have demonstrated that the USP22-FASN axis is highly activated in CRC and is critical for lipid accumulation and tumorigenesis.

## Results

### H_2_O_2_ inhibits fatty acid accumulation by promoting FASN ubiquitination and degradation in p53^+/+^ CRC cells

**Fig. 1:**
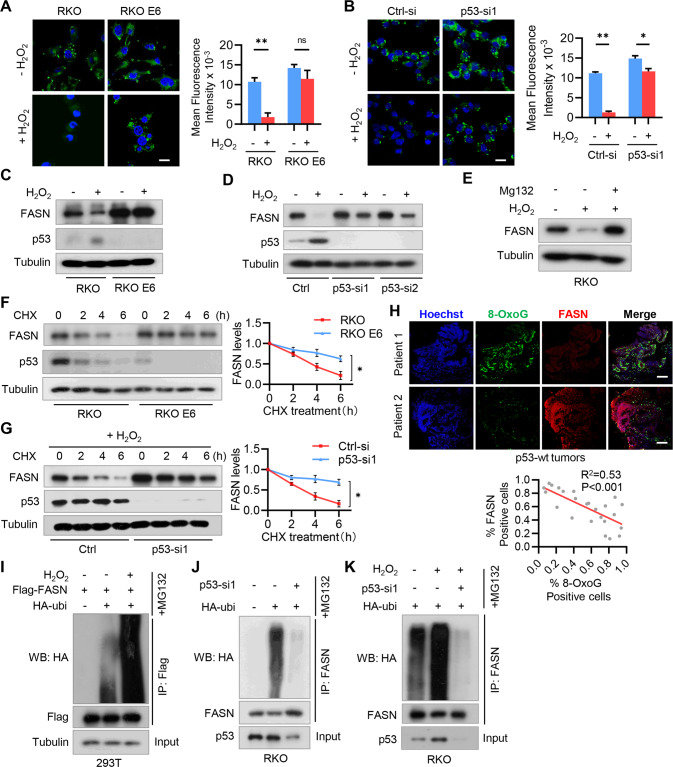
H_2_O_2_ downregulates lipid synthesis by promoting FASN ubiquitination and degradation in p53^+/+^ CRC cells. **A** RKO and RKO E6 CRC cells were treated with 100 µM H_2_O_2_ for 24 h, and cellular lipid droplets were visualized fluorescently with Bodipy 493/503. **B** RKO cells were transfected with siRNA against *p53*, and cellular lipid droplets were visualized fluorescently with Bodipy 493/503. In (**A**) and (**B**), representative images are shown. Scale bar, 20 µm. The fluorescence intensity of 20 randomly selected cells was quantified by ImageJ software. Data was expressed as 10^−3^ of the values of fluorescence intensity. ^*^P < 0.05, ^**^P < 0.001. ns not significant. **C** RKO and RKO E6 cells were treated with 100 µM H_2_O_2_ for 24 h, and cell lysates were analyzed by immunoblotting. **D** RKO cells were transfected with *p53* siRNAs and then treated with H_2_O_2_ for 24 h. Cell lysates were analyzed by immunoblotting. **E** RKO cells were treated with H_2_O_2_ for 36 h and then treated with MG132 for 6 h. Cell lysates were analyzed by immunoblotting. **F** RKO and RKO E6 cells were treated with CHX for the indicated time intervals and cell lysates were analyzed by immunoblotting. **G** RKO cells were transfected with *p53* siRNA and then treated with CHX for the indicated time intervals in the presence of H_2_O_2_. Cell lysates were analyzed by immunoblotting. In (**F**) and (**G**), band intensities of FASN were quantified and the results are expressed as FASN levels relative to the control cells (mean ± s.d., *n* = 3 independent experiments, paired Student’s t-test, right panels). ^∗^*P* < 0.01. **H** Human CRC specimens (p53 wild-type) were double stained with anti-8-OxoG and FASN antibodies. Representative images of two tumors are shown. Scale bar, 500 µm. The percentages of 8-OxoG and FASN positive cells in each microscope field were analyzed and compared (4 randomly selected fields for each tissue, 6 tissues, Pearson correlation test). **I** 293T cells were co-transfected with Flag-FASN and HA-Ubi plasmids, and then treated with H_2_O_2_ for 24 h. Cells were further treated with 20 µM MG132 for 6 h before harvest, and cell lysates were immunoprecipitated with an anti-Flag antibody and then analyzed by immunoblotting. **J** RKO cells were transfected with *p53* siRNA and HA-Ubi, and treated with 20 µM MG132 for 6 h. Cell lysates were immunoprecipitated with an anti-FASN antibody and then analyzed by immunoblotting. **K** RKO cells were transfected with *p53* siRNA and HA-Ubi, and then treated H_2_O_2_ for 24 h. Cell lysates were immunoprecipitated with an anti-FASN antibody and then analyzed by immunoblotting.

Both oxidative stress and aberrant lipid synthesis are established hallmarks of human cancer. To explore their regulatory relationship during tumorigenesis, we first assessed the effect of hydrogen peroxide (H_2_O_2_), the main endogenous ROS produced by mitochondria, on fatty acid (FA) accumulation in CRC cells. We found that cellular lipid droplets were sharply decreased in RKO cells (p53^+/+^) after H_2_O_2_ treatment (Fig. 1A). In contrast, H_2_O_2_ had no significant effect on lipid droplet formation in RKO E6 cells (p53^−/−^) (Fig. 1A). Accordingly, depletion of p53 attenuated the inhibitory effect of H_2_O_2_ on lipid droplet formation in RKO cells (Fig. 1B). These results suggest that H_2_O_2_ inhibits FA accumulation in a p53-dependent manner.

We next examined the effect of oxidative stress on the expression of FASN, a key enzyme responsible for the terminal catalytic step of *de novo* FA synthesis [11]. H_2_O_2_ has been reported to induce p53 expression [8, 19], which was confirmed in RKO cells (Fig. 1C, D and Fig. S1A). Compared with RKO E6 cells, FASN in RKO cells was significantly repressed by H_2_O_2_ (Fig. 1C and Fig. S1A). Accordingly, depletion of p53 reversed the inhibitory effect of H_2_O_2_ on FASN protein expression (Fig. 1D). However, H_2_O_2_ had no obvious effect on *FASN* mRNA expression (Fig. S1B). We thus supposed that H_2_O_2_ may regulate FASN expression through a proteasome-dependent pathway. To this end, we treated RKO cells with H_2_O_2_ in the presence of proteasome inhibitor MG132, and found MG132 reversed H_2_O_2_-induced FASN downregulation (Fig. 1E). Further, we found that FASN protein was more stable in RKO E6 cells than RKO cells (Fig. 1F). Accordingly, p53 depletion in RKO cells inhibited FASN degradation and reversed the inhibitory effect of H_2_O_2_ on FASN stability (Fig. 1G and Fig. S1C). To examine whether the levels of ROS and FASN are clinically relevant, we detected the levels of 8-oxoguanine (8-OxoG), indicative of oxidative DNA damage, and found FASN expression was negatively correlated with the 8-OxoG levels in p53 wild-type CRC specimens (Fig. 1H).

We next determined the effect of H_2_O_2_ on FASN ubiquitination, and found H_2_O_2_ treatment significantly increased FASN ubiquitination in the presence of proteasome inhibitor MG132 (Fig. 1I). Accordingly, depletion of p53 inhibits FASN ubiquitination in RKO cells (Fig. 1J). Notably, H_2_O_2_-induced ubiquitination of FASN was reversed by p53 depletion (Fig. 1K). Together, these results demonstrate that H_2_O_2_ destabilizes FASN by increasing its ubiquitination through a p53-dependent manner in colorectal cancer.

### USP22 interacts with FASN and inhibits FASN ubiqutination

**Fig. 2:**
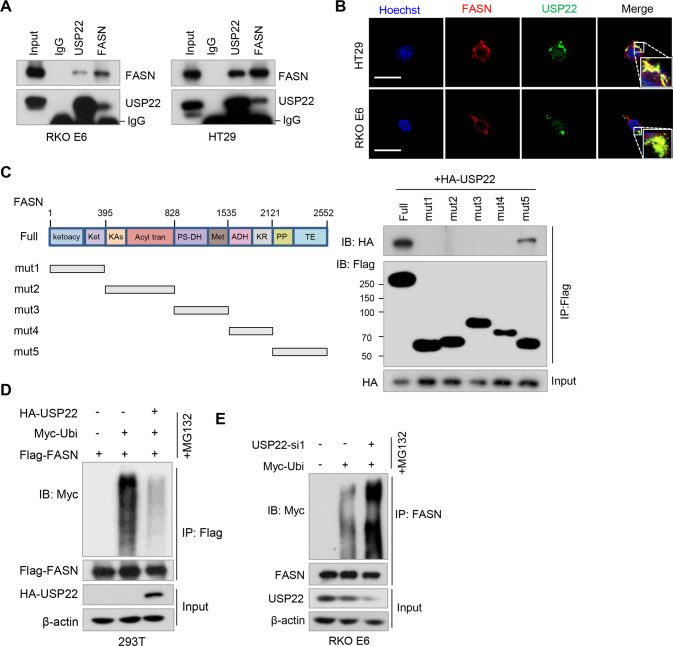
USP22 interacts with FASN and inhibits FASN ubiqutination. **A** RKO E6 and HT29 cell lysates were immunoprecipitated using antibodies against USP22 and FASN, and then subjected to immunoblotting. IgG was used as an isotype control. **B** Immunofluorescence assays were performed in RKO E6 and HT29 cells using antibodies against USP22 and FASN. Scale bar, 20 μM. Insets: high-magnification images. **C** A series of Flag-tagged FASN deletion mutants were constructed and co-transfected with HA-USP22 into 293T cells. Cell lysates were immunoprecipitated using an anti-Flag antibody and then analyzed by immunoblotting. The domains of FASN protein are indicated (left panel). **D** 293T cells were transfected with Flag-FASN, Myc-Ubi, and HA-USP22, and then treated with MG132 for 6 h. Cell lysates were immunoprecipitated using an anti-Flag antibody and then analyzed by immunoblotting. **E** RKO E6 cells were transfected with USP22 siRNA and Myc-Ubi, and then treated with MG132 for 6 h. Cell lysates were immunoprecipitated using an anti-FASN antibody and then analyzed by immunoblotting.

We supposed that H_2_O_2_ may regulate FASN ubiquitination and stability through a deubiquitinase (DUB). We, therefore, screened a panel of DUBs by transfecting the cDNA plasmids of 36 DUBs into 293T cells, and found USP2, USP14, USP22, and USP30 upregulated FASN levels (Fig. S2A). Of those DUBs, the effect of USP22 on FASN expression was most significant (Fig. S2A). Moreover, USP22 was the only DUB decreased by H_2_O_2_ treatment (Fig. S2B). We thus selected USP22 for further investigation. Reciprocal immunoprecipitation (IP) assays demonstrated that USP22 and FASN interact with each other in both RKO E6 and HT29 cells (Fig. 2A), which was further confirmed in 293T cells by co-transfection of HA-USP22 and Flag-FASN plasmids (Fig. S2C). Moreover, we found USP22 was expressed in both nucleus and cytoplasm, and partially co-localized with FASN in the cytoplasm in CRC cells (Fig. 2B). Further, using a series of Flag-tagged deletion mutants of FASN, we found that the Thioesterase (TE) domain in the C-terminal of FASN was required for its binding with USP22 (Fig. 2C).

We next assessed the effect of USP22 on FASN ubiquitination. Overexpression of USP22 decreased FASN ubiquitination in the presence of MG132 (Fig. 2D and Fig. S2D). Whereas, depletion of USP22 in RKO E6 cells promoted FASN ubiquitination (Fig. 2E). Together, these results support that USP22 interacts with FASN and inhibits FASN ubiquitination in CRC cells.

### H_2_O_2_ destabilizes FASN and represses lipid synthesis through USP22 in p53^+/+^ CRC cells

**Fig. 3:**
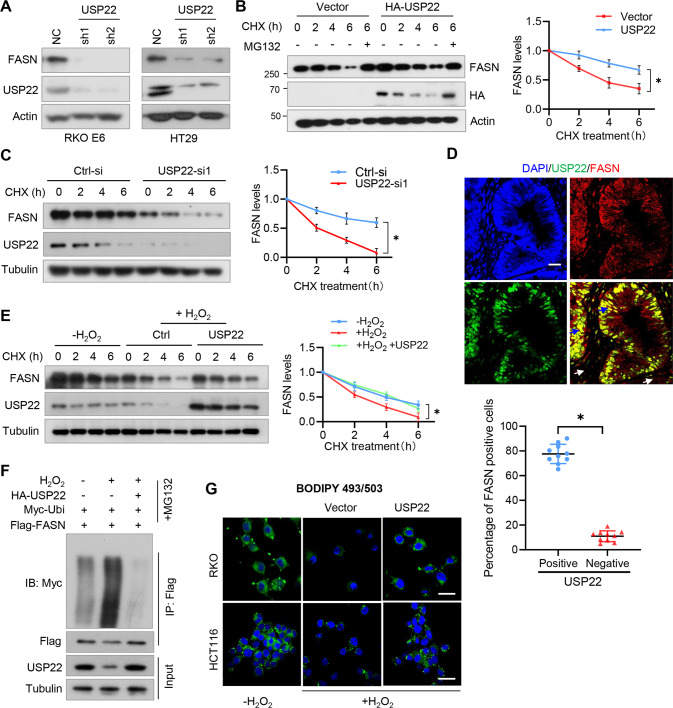
H_2_O_2_ destabilizes FASN and represses lipid synthesis through USP22 in p53^+/+^ CRC cells. **A** RKO E6 and HT29 cells expressing *USP22* shRNAs were lysed and then subjected to immunoblotting using FASN and USP22 antibodies. **B** 293T cells were transfected HA-USP22 or a control vector, and then treated with CHX and MG132 as indicated. Cell lysates were analyzed by immunoblotting. **C** RKO E6 cells expressing *USP22* shRNA were treated with CHX for the indicated time intervals. Cell lysates were analyzed by immunoblotting. **D** CRC tissues were double-stained with anti-USP22 and anti-FASN antibodies. Representative images are shown. The blue and white arrows indicate USP22/FASN double positive and double negative areas, respectively. Scale bars, 100 µm. In 10 randomly selected microscope fields of the tumor tissues, the percentages of USP22/FASN double-stained cells and USP22-negative but FASN-positive cells were analyzed and compared (mean ± s.e.m., unpaired Student’s t-test). **E** RKO cells were transfected with HA-USP22 and then treated with CHX for the indicated time intervals in the presence of H_2_O_2_ or not. Cell lysates were analyzed by immunoblotting. In **B**, **C**, and **E**, band intensities of FASN were quantified and the results are expressed as FASN levels relative to the control cells (mean ± s.d., *n* = 3 independent experiments, paired Student’s t-test, right panels). ^∗^*P* < 0.01. **F** 293T cells were transfected with HA-USP22, Myc-Ubi, and Flag-FASN, and then treated with H_2_O_2._ Cell lysates were immunoprecipitated using an anti-Flag antibody and then analyzed by immunoblotting. **G** RKO and HCT116 cells expressing USP22 were treated with H_2_O_2_ for 24 h. Cellular lipid droplets were visualized fluorescently with Bodipy 493/503. Representative images are shown. Scale bar, 20 µm.

We next determined the roles of USP22 in regulating FASN protein stability. As we expected, depletion of USP22 decreased the levels of FASN in CRC cells (Fig. 3A). While overexpression of USP22 stabilized FASN in 293T cells (Fig. 3B), USP22 depletion promoted FASN degradation in CRC cells under CHX treatment (Fig. 3C). In a panel of CRC cell lines, the expression levels of USP22 and FASN were positively correlated with each other (Fig. S3A). Accordingly, the depletion of USP22 significantly decreased lipid droplet formation in CRC cells (Fig. S3B). Further, using immunofluorescence-based co-expression assays, we found that the percentages of USP22/FASN double-stained cells were significantly higher than that of USP22-negative but FASN-positive cells in human CRC specimens (Fig. 3D), suggesting that FASN and USP22 are co-expressed in CRC cells.

To reveal the role of USP22 in regulating FASN expression under oxidative stress, 293T cells were transfected with USP22 and then treated with H_2_O_2_. We found that USP22 overexpression rescued the inhibitory effect of H_2_O_2_ on FASN stability (Fig. 3E). Mechanically, transfection of USP22 reversed the effect of H_2_O_2_ on FASN ubiqutination (Fig. 3F). Moreover, we found that overexpression of USP22 rescued the inhibitory effect of H_2_O_2_ on lipid accumulation in both RKO and HCT116 cells (Fig. 3G). Together, these results indicate that H_2_O_2_ regulates FASN ubiquitination and stability through USP22.

### p53 transcriptionally represses USP22 expression under H_2_O_2_ treatment

**Fig. 4:**
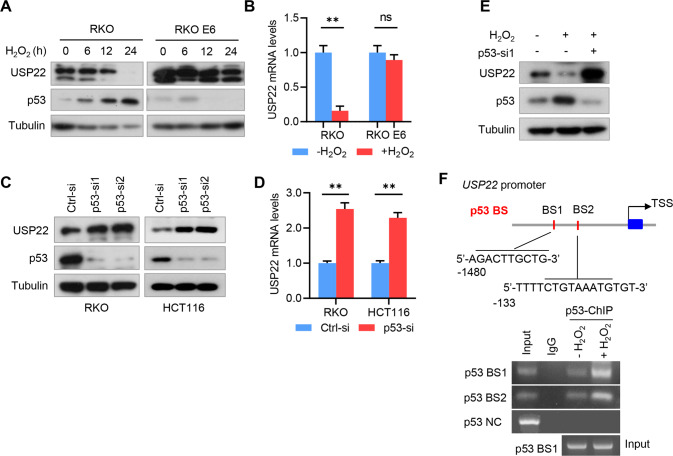
p53 transcriptionally represses USP22 expression under H_2_O_2_ treatment. **A** RKO and RKO E6 cells were treated H_2_O_2_ for the indicated time intervals, and cell lysates were subjected into immunoblotting using the indicated antibodies. **B** RKO and RKO E6 cells were treated H_2_O_2_ for 24 h, and *USP22* mRNA expression was examined by qRT-PCR. *GAPDH* was used as an internal control. Values were normalized to control (mean ± s.e.m., *n* = 3 independent experiments, two-tailed Student’s t-test). ns, not significant. ^**^P < 0.01. (**C**, **D**) RKO and HCT116 cells were transfected with siRNAs against *p53*, and USP22 protein and mRNA levels were analyzed by immunoblotting (**C**) and qRT-PCR (**D**), respectively. In **D**, values were normalized to control (mean ± s.e.m., *n* = 3 independent experiments, two-tailed Student’s t-test). ^**^P < 0.05. **E** RKO cells were transfected with *p53* siRNA, and then treated with H_2_O_2_ for 24 h. Cell lysates were subjected into immunoblotting using the indicated antibodies. **F** Chromatin immunoprecipitation (ChIP) assays demonstrate the binding of p53 to *USP22* promoter in RKO cells under H_2_O_2_ treatment. Two potential p53 binding elements were predicted by hTFtarget database and indicated. The resultant DNA from ChIP was analyzed by PCR and then separated by agarose gel electrophoresis. IgG was used as an isotype control, and an irrelevant DNA sequence was used as a negative control.

Because p53 is an important downstream effector mediating oxidative stress [19], and H_2_O_2_ inhibits FASN expression only in p53^+/+^ CRC cells (Fig. 1C, D), we hypothesized that H_2_O_2_ may regulate USP22 through p53. To this end, we treated RKO cells with H_2_O_2_, and found USP22 protein was dramatically repressed, and was negatively correlated with p53 expression (Fig. 4A and Fig. S4A). However, H_2_O_2_ had no obvious effect on USP22 expression in RKO E6 cells (Fig. 4A and Fig. S4A). The repression of USP22 by H_2_O_2_ should occur at the level of mRNA transcription, because H_2_O_2_ also inhibited *USP22* mRNA in RKO cells (Fig. 4B). To further validate the effect of p53 on USP22 expression, we depleted p53 in RKO and HCT116 cells, and found USP22 was increased by p53 depletion at both protein and mRNA levels (Fig. 4C, D). Notably, H_2_O_2_-induced downregulation of USP22 was reversed by the depletion of p53 (Fig. 4E and Fig. S4B).

We next determined whether p53 regulates USP22 expression by directly binding to the promoter region of *USP22*. ChIP-seq peaks in *USP22* promoter were analyzed by hTFtarget database (http://bioinfo.life.hust.edu.cn/hTFtarget/), and two potential p53 binding elements were predicted (Fig. 4F). We found that p53 specifically bound to those two sites, and their bindings were enhanced by H_2_O_2_ treatment (Fig. 4F). Together, these results support that H_2_O_2_ inhibits USP22 expression though p53-mediated transcription repression.

### H_2_O_2_ promotes cell cycle arrest and apoptosis by repressing the USP22-FASN pathway in p53^+/+^ CRC cells

**Fig. 5:**
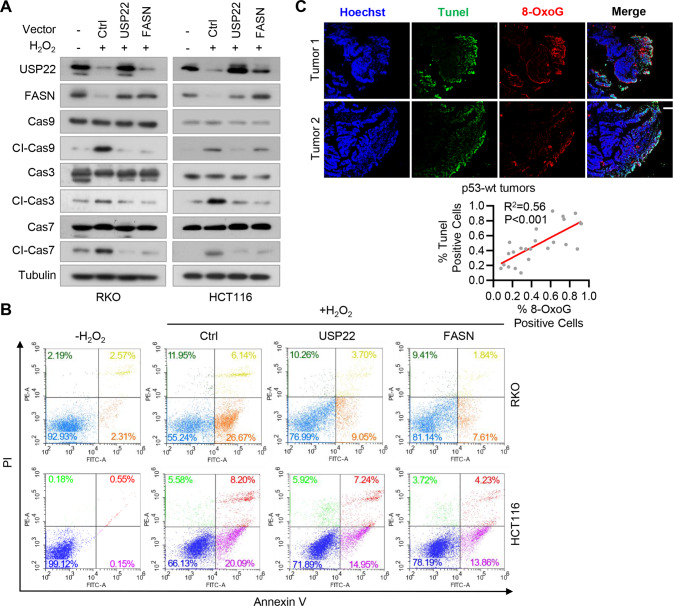
H_2_O_2_ promotes cell cycle arrest and apoptosis by repressing USP22-FASN pathway in p53^+/+^ CRC cells. **A** RKO and HCT116 cells stably expressing USP22 or FASN were treated with H_2_O_2_, and cell lysates were analyzed by immunoblotting using the indicated antibodies. **B** RKO and HCT116 cells expressing USP22 or FASN were treated with H_2_O_2_, and then double stained with Annexin V/propidium iodide (PI). Cell apoptosis was analyzed by flow cytometry. **C** CRC tissues with wild-type p53 were labeled by Tunel (Green) and anti-8-Oxoguanine antibody (Red), and images were taken using a Zeiss confocal microscope. Representative images of two tumors are shown. Scale bar, 200 μm. The percentages of 8-OxoG and Tunel positive cells in each microscope field were analyzed and compared (4 randomly selected fields for each tissue, 6 tissues, Pearson correlation test).

Because both USP22 and FASN are involved in the regulation of cell apoptosis [20, 21], we next determined the role of the USP22-FASN axis in H_2_O_2_-induced cell apoptosis in p53^**+/+**^ CRC cells. H_2_O_2_ treatment downregulated USP22 and FASN in RKO and HCT116 cells (Fig. 5A), but induced the expression of cleaved CASP3/7 and CASP9, which is consistent with the results by others [22]. We found that reconstituted expression of USP22 or FASN reversed the effect of H_2_O_2_ on the expression of cleaved CASP3/7 and CASP9 (Fig. 5A). Accordingly, flow cytometry analysis of cells stained with PI and Annexin V showed that H_2_O_2_ induced RKO and HCT116 cell apoptosis, and exogenous USP22 or FASN reversed the stimulative effect of H_2_O_2_ on cell apoptosis (Fig. 5B). Moreover, the level of 8-OxoG, indicative of oxidative DNA damage, was positively correlated with the extent of cell apoptosis in p53 wild-type CRC tissues (Fig. 5C). These results suggest that H_2_O_2_ induces cells apoptosis by repressing the USP22-FASN axis in p53^**+/+**^ colorectal cancer cells.

### Depletion of the USP22-FASN axis inhibits cell growth and colorectal tumorigenesis

**Fig. 6:**
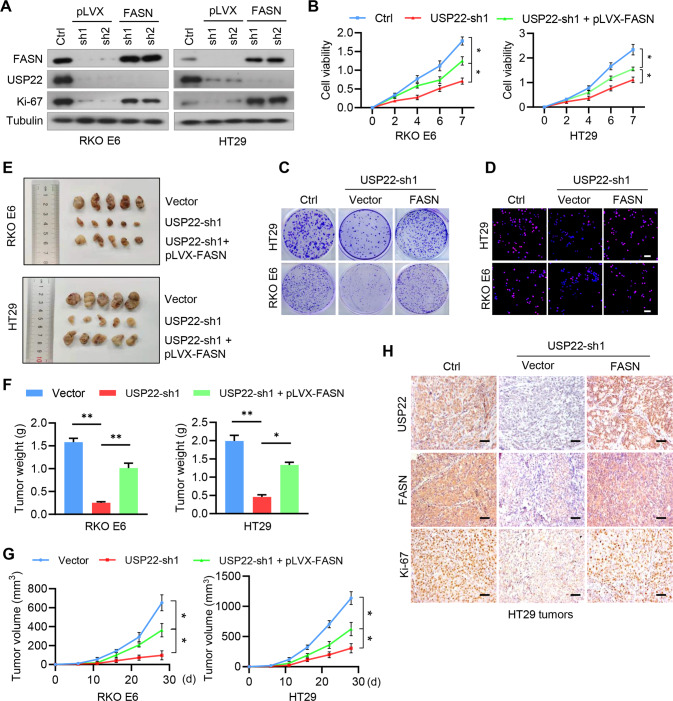
Depletion of the USP22-FASN axis inhibits cell proliferation and colorectal tumorigenesis. **A** RKO E6 and HT29 cells stably expressing *USP22* shRNAs were reconstituted by the expression of FASN, and cell lysates were subjected to immunoblotting using the indicated antibodies. **B** RKO E6 and HT29 cells stably expressing *USP22* shRNAs were reconstituted by the expression of FASN, and cell viability was assessed by CCK8 (mean ± s.e.m., *n* = 3 independent experiments, paired Student’s t-test). ^*^P < 0.01. **C** Cell growth of RKO E6 and HT29 cells was measured by clony-formation assays. Representative images were shown. **D** Cell proliferation of RKO E6 and HT29 cells was detected using the BrdU assays. Representative images were shown. Scale bar, 200 μm. **E**–**G** RKO E6 and HT29 cells (5 × 10^6^ cells per mouse) stably expressing control shRNA, *USP22* shRNA, or *USP22* shRNA+pLVX-FASN were subcutaneously injected into nude mice. Four weeks after injection, the mice were humanely killed. The resected tumors of each group were shown (**E**), the tumor volume for each time point after injection was calculated (**F**), and the tumor weight was recorded (**G**). Data was expressed as mean ± s.d., *n* = 5 mice for each group, one-way ANOVA test, *P < 0.01. ^**^P < 0.001. **H** Consecutive sections from tumor xenografts derived from HT29 cells were immunostained using antibodies against USP22, FASN, and Ki-67, respectively. Representative microphotographs of immunostaining for each group are shown. Scale bars, 200 µm.

We next investigated the role of USP22-mediated stabilization of FASN in CRC cell proliferation and tumor growth. Depletion of USP22 repressed the expression of the cell proliferation antigen Ki-67, and reconstituted expression of FASN rescued this effect (Fig. 6A). Accordingly, USP22 depletion inhibited cell viability and colony formation in both HT29 and RKO E6 cells, and exogenous FASN reversed the effect of USP22 depletion on cell growth (Fig. 6B, C). The results were further validated by BrdU assays (Fig. 6D). Using a subcutaneous implant mouse model, we next evaluated whether USP22 regulates colorectal tumorigenesis through FASN. We found that USP22 depletion in HT29 and RKO E6 cells attenuated tumor formation, and reconstituted expression of FASN rescued the inhibitory effect of USP22 depletion on colorectal tumorigenesis (Fig. 6E–G). Moreover, the depletion of USP22 decreased the levels of FASN and Ki-67 in mouse xenograft tumors, and exogenous FASN rescued those effects (Fig. 6H). Together, these results support that USP22 promotes CRC cell proliferation and tumorigenesis by stabilizing FASN.

### USP22 is positively correlated with FASN expression, and high USP22/FASN levels predict poor prognosis in colorectal cancer

**Fig. 7:**
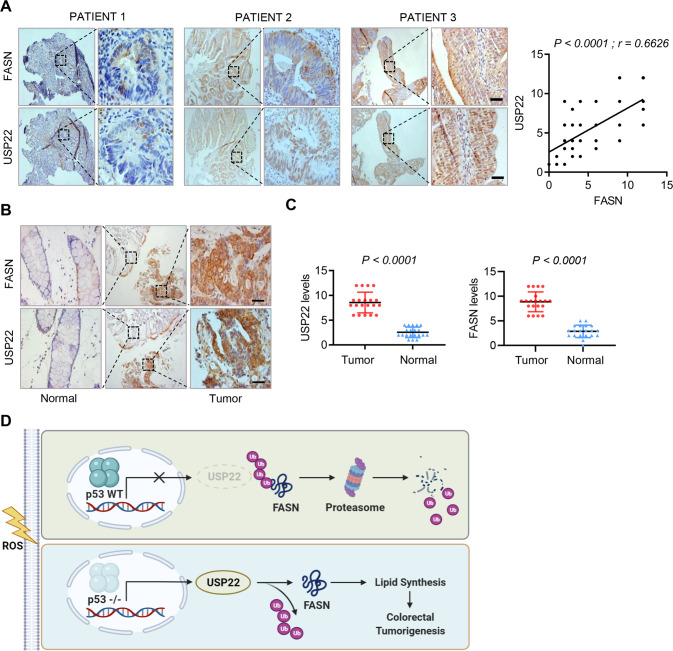
FASN is positively correlated with USP22 expression in colorectal cancer. **A** Immunostaining of USP22 and FASN in human CRC specimens. Representative images of three tumors are shown. Insets: high-magnification images corresponding to the areas marked by black dotted lines. Scale bars, 100 µm. Staining of USP22 and FASN was scored on a scale of 1–12. The correlation between USP22 and FASN was statistically significant among different specimens (*n* = 108 colorectal tumors, top panel, *r* = 0.6626, *P* < 0.001). Note that the scores of some samples overlap (right panel). **B**, **C** Human CRC tissues and the paired paracancerous non-tumor tissues were immunostained with anti-USP22 and anti-FASN antibodies, respectively. Representative images of the immunostaining were shown (**B**). Scale bars, 200 µm. The expression scores of USP22 and FASN were compared in the tumor and non-tumor tissues (*n* = 20 CRC tissues, paired Student’s t-test). **D** Diagram shows the findings revealed in this study. In p53 wild-type CRCs, ROS-induced p53 expression represses the transcription of USP22, which otherwise deubiquitinates and stabilizes FASN, and thus inhibits FA synthesis and tumor growth. In p53-deficent tumors, ROS-mediated inhibition of USP22 is relieved, leading to FASN stabilization, which thus promotes lipid synthesis and tumorigenesis.

Due to the high-frequency of p53 mutation in CRC, *USP22* mRNA should be upregulated in CRC tissues compared with normal tissues, which was confirmed by analysis of GEO dataset (Fig. S5A). Moreover, high-level *USP22* predicted worse overall and disease-free survivals in colorectal cancer (Fig. S5B). To determine the potential clinical relevance of the USP22-FASN axis, we next assessed the expression of USP22 and FASN proteins in serial sections of 108 human CRC specimens, and found that FASN expression levels were significantly correlated with USP22 levels (Fig. 7A, *r* = 0.6626, *P* < 0.0001). Moreover, both USP22 and FASN were significantly upregulated in CRC tumor tissues compared with the paired paracancerous non-tumor tissues (Fig. 7B, C). Together, these results suggest that FASN is stabilized by USP22 in colorectal cancer, and the dysregulated USP22/FASN axis is a important driver for tumorigenesis.

## Discussion

In this study, we have demonstrated that ROS in colorectal cancer critically regulates lipid synthesis through a p53-dependent mechanism. We have found H_2_O_2_-induced p53 expression inhibits the transcription of USP22, which otherwise deubiquitinates and stabilizes FASN. Whereas, in p53-deficient CRC cells, relief of ROS-mediated USP22 repression promotes FASN stabilization and lipid accumulation, and thus promotes tumorigenesis (Fig. 7D). Our findings establish that the USP22-FASN axis plays an important role in regulating lipid accumulation and colorectal tumorigenesis, and targeting this axis may represent a promising therapeutic strategy for colorectal cancer.

Tumor cells frequently display higher ROS production compared to healthy cells, and numerous studies have been dedicated to delineate when ROS are oncogenic and when they are tumor suppressive. It is believed that low to moderate level of ROS may contribute to cell proliferation and tumor growth, and high level of ROS promotes cell death [23]. However, growing evidence has supported that cellular response to ROS is context dependent [24–26]. Moreover, recent reports also suggest that the role of ROS in tumor development is stage specific. ROS is required for early stages but suppresses later stages of cancer development [27, 28]. Our study reveals that ROS inhibits FASN-mediated lipid synthesis in a p53-dependent manner, and demonstrates a novel mechanism for p53 in determining ROS-mediated cell response during tumorigenesis.

Tumor suppressor p53 plays an important role in lipid metabolism. Wild-type p53 enhances fatty acid oxidation while inhibiting fatty acid synthesis. Whereas, mutant p53 exert its function oppositely [29]. For instance, p53 induces the expression of carnitine palmitoyltransferase 1C (CPT1C), a key enzyme for mitochondria transport of activated fatty acids during lipid β-oxidation, and thus protects cells from metabolic stress-induced death [30]. Further, wild-type p53 represses the transcriptional activity of sterol response element-binding protein (SREBP1) [31], while mutant p53 enhances its function, leading to increased lipid synthesis in tumors [32]. Our study demonstrates that p53 represses USP22-mediated stabilization of FASN, thus provides an alternative pathway connecting p53 and fatty acid synthesis. Because p53 mutation usually loss the transcriptional activity and responses to upstream signals differently [33, 34], p53-mediated repression of the USP22-FASN axis will be abolished in p53-mutated cancers, resulting in FASN stabilization, lipid accumulation, and tumor growth.

As a critical enzyme catalyzing the synthesis of fatty acids, FASN is overexpressed in multiple cancers and required for cancer stem cell (CSC) maintenance, tumor initiation and progression [12]. Several deubiquitinases including USP14 and USP2 have been identified to regulate FASN deubiqtuination and stability [13, 35], which were confirmed in our study by detecting their effect on FASN expression (Fig.S2A). However, of those Dubs that increase FASN protein levels, only USP22 is repressed by H_2_O_2_ (Fig.S2B), indicating a specific role of USP22 in mediating H_2_O_2_-induced cellular responses. We and others have demonstrated that USP22 is upregulated in multiple types of CSCs, and controls CSC self-renewal and tumor chemoresistance [36, 37]. Thus, the USP22-FASN axis revealed in this study may be a critical mechanism for the maintenance of CSC properties and therapy resistance of human cancer, which deserves further investigation.

Recent reports about ROS on lipid synthesis are usually inconsistent [15–18], and the effect of elevated ROS production on lipid synthesis in tumor is almost unknown. Our study demonstrates that overproduced ROS in colorectal cancer controls lipid synthesis by critically regulating the USP22-FASN axis through a p53-dependent manner. Due to a high-frequency of p53 mutation and dysfunction in human cancer, activation of the USP22-FASN axis may represent a prevailing mechanism that drives tumorigenesis, indicating a promising strategy for the treatment of colorectal cancer.

## Materials and methods

### Cell culture, transfection, and treatment

Human colorectal cancer cell lines RKO, RKO E6, HCT116, Caco2, SW480, SW620, HT29, FHC, NCM460, and the embryonic kidney (HEK) 293T and 293FT cells were cultured in DMEM medium supplemented with 10% bovine calf serum (Gibco). No cell lines used in this study were found in the database of commonly misidentified cell lines that is maintained by ICLAC and NCBI Biosample. Cell lines were authenticated by short tandem repeat profiling and were routinely tested for mycoplasma contamination.

Transfections of plasmids and siRNAs were performed using lipofectamine 2000 (Invitrogen, CA, USA) and X-tremeGENE siRNA transfection reagents (Roche, Mannheim, Germany), respectively. The standard concentrations of the following reagent for cell treatment are: H_2_O_2_, 100 µM; cycloheximide (CHX), 50 µg/ml; MG132, 20 µM.

### Antibodies, reagent, and siRNAs

Detailed information about antibodies used in this study are shown in Table S1. Cycloheximide (CHX), MG132, H_2_O_2_ were from Sigma. Puromycin and Neomycin were EMD Biosciences (La Jolla, CA, USA). All siRNAs were synthesized from Genepharma (Shanghai, PRC), and the target sequences of the siRNAs are shown in Table S2.

### Plasmid construction

Flag-FASN plasmid was kindly provided by Dr. Dan Ye (Fudan University). The cDNA of FASN truncation mutants was amplified from Flag-FASN by PCR and cloned into pcDNA3.1-Flag vector. HA-USP22 and other DUBs were kept in the lab. Full length cDNAs of human *FASN* and *USP22* were also subcloned into the pLVX-neo vector. Plasmids expressing *USP22* shRNAs were constructed by annealing the sense and antisense oligonucleotides of the target sequence and cloned into the pLKO.1-puro vector. All plasmids were confirmed by DNA sequencing. Table S2 contains detailed information about the sequence of oligonucleotides.

### Lentiviral transfection and selection

The lentiviral expression plasmids, packaging plasmid psPAX2, and envelope plasmid pMD2.G (4:3:1) were co-transfected into 293FT cells using Lipofectamine 2000 reagent (Invitrogen). The cell culture media was changed 8 h after transfection, and lentivirus-containing medium was collected 24 h later. CRC cells in six-well plates were infected by adding the lentivirus and then selected by Puromycin (2 µg/ml) or Neomycin (800 µg/ml) for 1 week.

### ChIP

Chromatin immunoprecipitation (ChIP) assays were performed using a SimpleChIP^®^ Enzymatic Chromatin IP Kit (Cell Signaling Technology), accordingly to the manufacturer’s instructions. Briefly, cells were crosslinked with 1% formaldehyde for 10 min at room temperature and then quenched with 125 mM glycine. The isolated nuclei were resuspended in nuclei lysis buffer and then pulse sonicated. The samples were immunoprecipitated with 5 μg of anti-p53 antibody overnight at 4 °C, and then incubated with protein A/G beads for 1 h. The immunoprecipitates were washed with low salt, high salt, and LiCl buffers, and the immunoprecipitated DNA was reverse-crosslinked and purified. The resultant DNA was analyzed by semi-qRT-PCR and determined by electrophoresis in a 2% agarose gel. Primer used for PCR are shown in Table S2.

### BODIPY 493/503 staining

Cellular lipid droplets were stained with Bodipy 493/503 (ThermoFisher, MA, USA) accordingly to the manufacturer’s instruction. Briefly, cells were washed twice with PBS, and then incubated with appropriate amount of BODIPY 493/503 working solution (2 µM in PBS) at 37 °C for 15 min. Cells were fixed with 4% paraformaldehyde at room temperature for 30 min and then incubated with Hoechst solution for 10 min. The slides were mounted with anti-fluorescence quencher, and images were taken using a confocal microscope (Carl Zeiss).

### Immunofluorescence and immunohistochemistry

For immunofluorescence (IF) analysis, cells in six-well plates were treated with 4% formaldehyde and then treated with 0.5% Triton X-100 for 5 min. The slides were incubated with antibodies against USP22 and FASN, and then incubated with a fluorescent-conjugated second antibody (Alexa Fluor, Life Technologies, 1:500). Nuclei were co-stained with DAPI. Images were taken using a confocal microscope (Carl Zeiss).

Anonymous archived human CRC specimens were obtained from the Department of Gastroenterology of Nanfang Hospital of Sourthern Medical University under a protocol approved by the institutional review board. All tissue samples were collected in compliance with the institution’s informed consent policy. For immunohistochemical (IHC) staining, tissue slides were deparaffinized, rehydrated through an alcohol series, and then stained with primary antibodies against USP22, FASN and Ki-67. We quantified the scores of USP22 and FASN staining according to the percentage of cells with positive staining and the staining intensity. We assigned the percentage score as follows: 0 if no cell had staining, 1 if 0–25% of cells had staining, 2 if 25–50%, 3 if 50–75%, 4 if more than 75% of cells had staining. We scored the staining intensity as 0 for negative, 1 for weak, 2 for moderate, and 3 for strong. The total score was obtained by multiplying the percentage score by the intensity score. Three individuals who were blinded to the slides examined and scored each sample. The final score was the median value of the scores provided by the individuals.

### Fluorescence detection of 8-oxoguanine

We detected the levels of 8-oxoguanine (8-OxoG), the major DNA lesions formed from ROS, in human CRC specimens using an anti-8-Oxoguanine antibody (Bioss, Beijing, China). The detailed procedure of this experiment is as we described above in Immunofluorescence.

### Cell growth assays

For cell growth analysis, CRC cells (1000 cells/well) were resuspended in DMEM and cultured at 37 °C in a humidified CO_2_ incubator for 10 days. Colonies were stained with 0.05% crystal violet, and those that were >1 mm in diameter were counted. The viability of cells was assessed by CCK8 assays (Vazyme Biotech), according to the manufacturer’s instructions.

### Cellular ubiquitination assays

Cellular ubiquitination assays were performed as we described previously [36]. Briefly, cells were transfected with the indicated plasmids and treated with the proteasome inhibitor MG132 (20 µM) for 6 h before harvest. Cells were lysed using RIPA lysis buffer (50 mM Tris-base pH 6.8, 150 mM NaCl, 1% NP-40, 0.5% deoxycholic acid, 0.1% SDS, 10 mM NaF, 10 mM dithiothreitol (DTT), 0.2 mM Na_3_VO_4_, 1% cocktail protease inhibitors, 1 mM phenylmethylsulfonyl fluoride (PMSF)). Cell lysates were immunoprecipitated using the indicated antibodies and washed three times by RIPA buffer. To exclude nonspecific ubiquitin-modified species from the FASN complex, we washed the immunoprecipitates three times using a ubiquitylation wash buffer (50 mM Trisbase pH 6.8, 150 mM NaCl, 1% NP-40, 0.5% deoxycholic acid, 1 M urea, 1 mM Nethylmaleimide (NEM), and protease inhibitors). The resultant precipitates were separated by SDS page and then subjected to immunoblotting.

### In vivo subcutaneous xenograft mouse model

All mouse experiments were approved by the Institutional Animal Care and Use Committee of Southern Medical University. The sample sizes were justified by statistical considerations and statistical power analyses. The animals were randomly assigned to different experimental groups. The investigators were blinded to allocation during experiments and outcome assessment. RKO E6 and HT29 cells (5 × 10^6^ cells/mouse) expressing the indicated shRNAs or proteins were injected subcutaneously into 6- to 8-week-old nude (nu/nu) mice. Tumor volumes were calculated using the formula V = (π/6) × *a*^2^ × *b*, where a and b are the tumor’s short axis and long axis, respectively. At the end of the experiment, the mice were humanely killed, and tumor tissues was harvested, weighed, and then fixed in 4% formaldehyde and embedded in paraffin.

### Statistical analyses

GraphPad Prism 8.0 software was used for all data analysis. Data are presented as the means ± standard deviation (s.d.) or standard errors of the mean (s.e.m.). All western blot experiments were repeated three times unless otherwise indicated. For all representative images, results were reproduced at least three times in independent experiments. For all quantitative data, the statistical test used is indicated in the figure legends. We assessed differences in the human colorectal cancer data using the Pearson correlation test, the in vitro data between two groups (=2 groups) using the two-tailed Student’s t-test, the in vitro data among multiple groups (>2 groups), and the in vivo data using two-way analysis of variance (ANOVA). We considered *P* < 0.05 to be significant.

## Supplementary information

Supplementary Information

Original Data File

Agreement of authorship changes

## Data Availability

All data that support the conclusions are available from the authors on reasonable request, and/or available in the manuscript itself.

## References

[1] Hayes JD, Dinkova-Kostova AT, Tew KD (2020). Oxidative stress in cancer. Cancer Cell.

[2] Perillo B, Di Donato M, Pezone A, Di Zazzo E, Giovannelli P, Galasso G (2020). ROS in cancer therapy: The bright side of the moon. Exp Mol Med.

[3] Wang J, Luo B, Li X, Lu W, Yang J, Hu Y (2017). Inhibition of cancer growth in vitro and in vivo by a novel ROS-modulating agent with ability to eliminate stem-like cancer cells. Cell Death Dis.

[4] Duffy MJ, Synnott NC, O’Grady S, Crown J. Targeting p53 for the treatment of cancer. Semin Cancer Biol*.* 2020;79:58–67. 10.1016/j.semcancer.2020.07.005 32741700

[5] Nakayama M, Oshima M (2019). Mutant p53 in colon cancer. J Mol Cell Biol.

[6] Meng Q, Shi S, Liang C, Liang D, Hua J, Zhang B (2018). Abrogation of glutathione peroxidase-1 drives EMT and chemoresistance in pancreatic cancer by activating ROS-mediated Akt/GSK3beta/Snail signaling. Oncogene.

[7] Zhao Y, Hu X, Liu Y, Dong S, Wen Z, He W (2017). ROS signaling under metabolic stress: Cross-talk between AMPK and AKT pathway. Mol Cancer.

[8] Samuel J, Jayne S, Chen Y, Majid A, Wignall A, Wormull T (2016). Posttranscriptional upregulation of p53 by reactive oxygen species in chronic lymphocytic leukemia. Cancer Res.

[9] Cordani M, Butera G, Pacchiana R, Masetto F, Mullappilly N, Riganti C, et al. Mutant p53-associated molecular mechanisms of ROS regulation in cancer cells. Biomolecules. 2020;10:361. 10.3390/biom10030361 PMC7175157 32111081

[10] Koundouros N, Poulogiannis G (2020). Reprogramming of fatty acid metabolism in cancer. Br J Cancer.

[11] Rohrig F, Schulze A (2016). The multifaceted roles of fatty acid synthesis in cancer. Nat Rev Cancer.

[12] Bacci M, Lorito N, Smiriglia A, Morandi A (2021). Fat and furious: Lipid metabolism in antitumoral therapy response and resistance. Trends Cancer.

[13] Liu B, Jiang S, Li M, Xiong X, Zhu M, Li D (2018). Proteome-wide analysis of USP14 substrates revealed its role in hepatosteatosis via stabilization of FASN. Nat Commun.

[14] Berkers CR, Maddocks OD, Cheung EC, Mor I, Vousden KH (2013). Metabolic regulation by p53 family members. Cell Metab.

[15] Liu L, MacKenzie KR, Putluri N, Maletic-Savatic M, Bellen HJ (2017). The Glia-neuron lactate shuttle and elevated ROS promote lipid synthesis in neurons and lipid droplet accumulation in glia via APOE/D. Cell Metab.

[16] Seo E, Kang H, Choi H, Choi W, Jun HS (2019). Reactive oxygen species-induced changes in glucose and lipid metabolism contribute to the accumulation of cholesterol in the liver during aging. Aging Cell.

[17] Douglas DN, Pu CH, Lewis JT, Bhat R, Anwar-Mohamed A, Logan M (2016). Oxidative stress attenuates lipid synthesis and increases mitochondrial fatty acid oxidation in hepatoma cells infected with hepatitis C virus. J Biol Chem.

[18] Islam A, Kagawa Y, Miyazaki H, Shil SK, Umaru BA, Yasumoto Y (2019). FABP7 protects astrocytes against ROS toxicity via lipid droplet formation. Mol Neurobiol.

[19] Montero J, Dutta C, van Bodegom D, Weinstock D, Letai A (2013). p53 regulates a non-apoptotic death induced by ROS. Cell Death Differ.

[20] Lin ZH, Yang H, Kong QF, Li JP, Lee SM, Gao BX (2012). USP22 antagonizes p53 transcriptional activation by deubiquitinating Sirt1 to suppress cell apoptosis and Is required for mouse embryonic development. Mol Cell.

[21] Al-Bahlani S, Al-Lawati H, Al-Adawi M, Al-Abri N, Al-Dhahli B, Al-Adawi K (2017). Fatty acid synthase regulates the chemosensitivity of breast cancer cells to cisplatin-induced apoptosis. Apoptosis.

[22] Rahman A, Pallichankandy S, Thayyullathil F, Galadari S (2019). Critical role of H2O2 in mediating sanguinarine-induced apoptosis in prostate cancer cells via facilitating ceramide generation, ERK1/2 phosphorylation, and Par-4 cleavage. Free Radic Biol Med.

[23] Moloney JN, Cotter TG (2018). ROS signalling in the biology of cancer. Semin Cell Dev Biol.

[24] Chen F, Su R, Ni S, Liu Y, Huang J, Li G (2021). Context-dependent responses of Drosophila intestinal stem cells to intracellular reactive oxygen species. Redox Biol.

[25] Sies H, Jones DP (2020). Reactive oxygen species (ROS) as pleiotropic physiological signalling agents. Nat Rev Mol Cell Biol.

[26] Kalo E, Kogan-Sakin I, Solomon H, Bar-Nathan E, Shay M, Shetzer Y (2012). Mutant p53R273H attenuates the expression of phase 2 detoxifying enzymes and promotes the survival of cells with high levels of reactive oxygen species. J Cell Sci.

[27] Piskounova E, Agathocleous M, Murphy MM, Hu Z, Huddlestun SE, Zhao Z (2015). Oxidative stress inhibits distant metastasis by human melanoma cells. Nature.

[28] Bagati A, Moparthy S, Fink EE, Bianchi-Smiraglia A, Yun DH, Kolesnikova M (2019). KLF9-dependent ROS regulate melanoma progression in stage-specific manner. Oncogene.

[29] Assaily W, Rubinger DA, Wheaton K, Lin Y, Ma W, Xuan W (2011). ROS-mediated p53 induction of Lpin1 regulates fatty acid oxidation in response to nutritional stress. Mol Cell.

[30] Sanchez-Macedo N, Feng J, Faubert B, Chang N, Elia A, Rushing EJ (2013). Depletion of the novel p53-target gene carnitine palmitoyltransferase 1C delays tumor growth in the neurofibromatosis type I tumor model. Cell Death Differ.

[31] Yahagi N, Shimano H, Matsuzaka T, Najima Y, Sekiya M, Nakagawa Y (2003). p53 Activation in adipocytes of obese mice. J Biol Chem.

[32] Freed-Pastor WA, Mizuno H, Zhao X, Langerod A, Moon SH, Rodriguez-Barrueco R (2012). Mutant p53 disrupts mammary tissue architecture via the mevalonate pathway. Cell.

[33] Sullivan KD, Galbraith MD, Andrysik Z, Espinosa JM (2018). Mechanisms of transcriptional regulation by p53. Cell Death Differ.

[34] Li G, Wu J, Li L, Jiang P. p53 deficiency induces MTHFD2 transcription to promote cell proliferation and restrain DNA damage. Proc Natl Acad Sci USA. 2021;118:e2019822118. 10.1073/pnas.2019822118 PMC8285905 34244426

[35] Graner E, Tang D, Rossi S, Baron A, Migita T, Weinstein LJ (2004). The isopeptidase USP2a regulates the stability of fatty acid synthase in prostate cancer. Cancer Cell.

[36] Zhou AD, Lin KY, Zhang SC, Chen YH, Zhang N, Xue JF (2016). Nuclear GSK3 beta promotes tumorigenesis by phosphorylating KDM1A and inducing its deubiquitylation by USP22. Nat Cell Biol.

[37] Ling S, Shan Q, Zhan Q, Ye Q, Liu P, Xu S (2020). USP22 promotes hypoxia-induced hepatocellular carcinoma stemness by a HIF1alpha/USP22 positive feedback loop upon TP53 inactivation. Gut.

