# Supplementary material for: Derepression of the USP22-FASN axis by p53 loss under oxidative stress drives lipogenesis and tumorigenesis
Source: Cell Death Discov. 2022 Nov 4;8:445. doi: 10.1038/s41420-022-01241-9 (PMC9636132; doi:10.1038/s41420-022-01241-9)
Supplement: Supplementary file 1 — Supplementary Information [file 41420_2022_1241_MOESM1_ESM.docx]

***Supplementary Information***

**Derepression of the USP22-FASN Axis by p53 Loss under Oxidative Stress Drives Lipogenesis and Tumorigenesis**

Zelong Han, Ming Liu, Yuxin Xie, Kunlin Zeng, Ziling Zhan, Yanwen Chen, Li Wang, Xiaoxia Chen, Yaxin Luo, Yu Zeng, Hongchao Zhan, Yingzhuo Lin, Keqin Zhang, Xiaoxia Zhu, Side Liu, Xiaobei Luo, Aidong Zhou

The Supporting Information contains 5 Supplementary Figures and 2 tables.


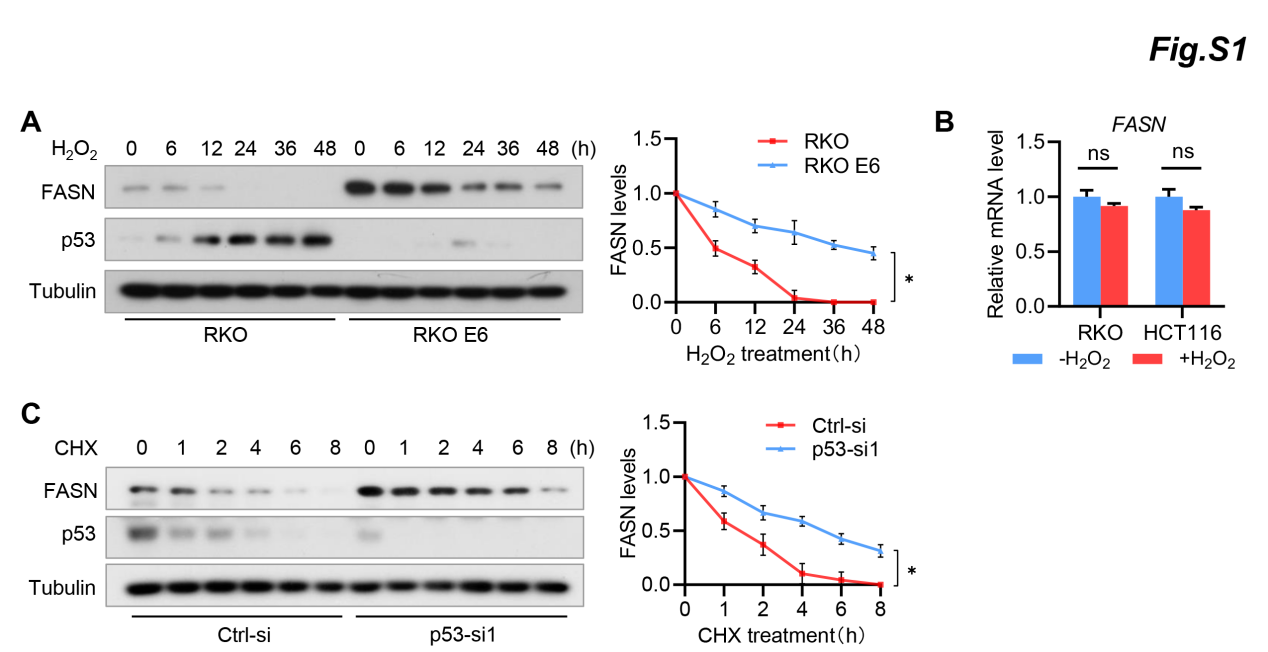


**Figure S1** H_2_O_2_ promotes FASN degradation in a p53-dependent manner.

**(A)** RKO and RKO E6 CRC cells were treated with 100µM H_2_O_2_ for the indicated time intervals, and cell lysates were analyzed by immunoblotting using the indicated antibodies. Band intensities of FASN were quantified and the results are expressed as FASN levels relative to untreated cells (mean ± s.d., n=3 independent experiments, paired Student’s t-test, right panel). ^∗^P < 0.01.

**(B)** RKO and HCT116 cells were treated with 100µM H_2_O_2_ for 36h, and *FASN* mRNA levels were determined by qRT-PCR. Values were normalized to that in control cells. *GAPDH* was used as internal control. ns, not significant.

**(C)** RKO cells transfected with control siRNA or *p53* siRNA were treated with CHX for the indicated time intervals, and cells lysates were analyzed by immunoblotting using the indicated antibodies. Band intensities of FASN were quantified and the results are expressed as FASN levels relative to untreated cells (mean ± s.d., n=3 independent experiments, paired Student’s t-test, right panel). ^∗^P < 0.01.

**
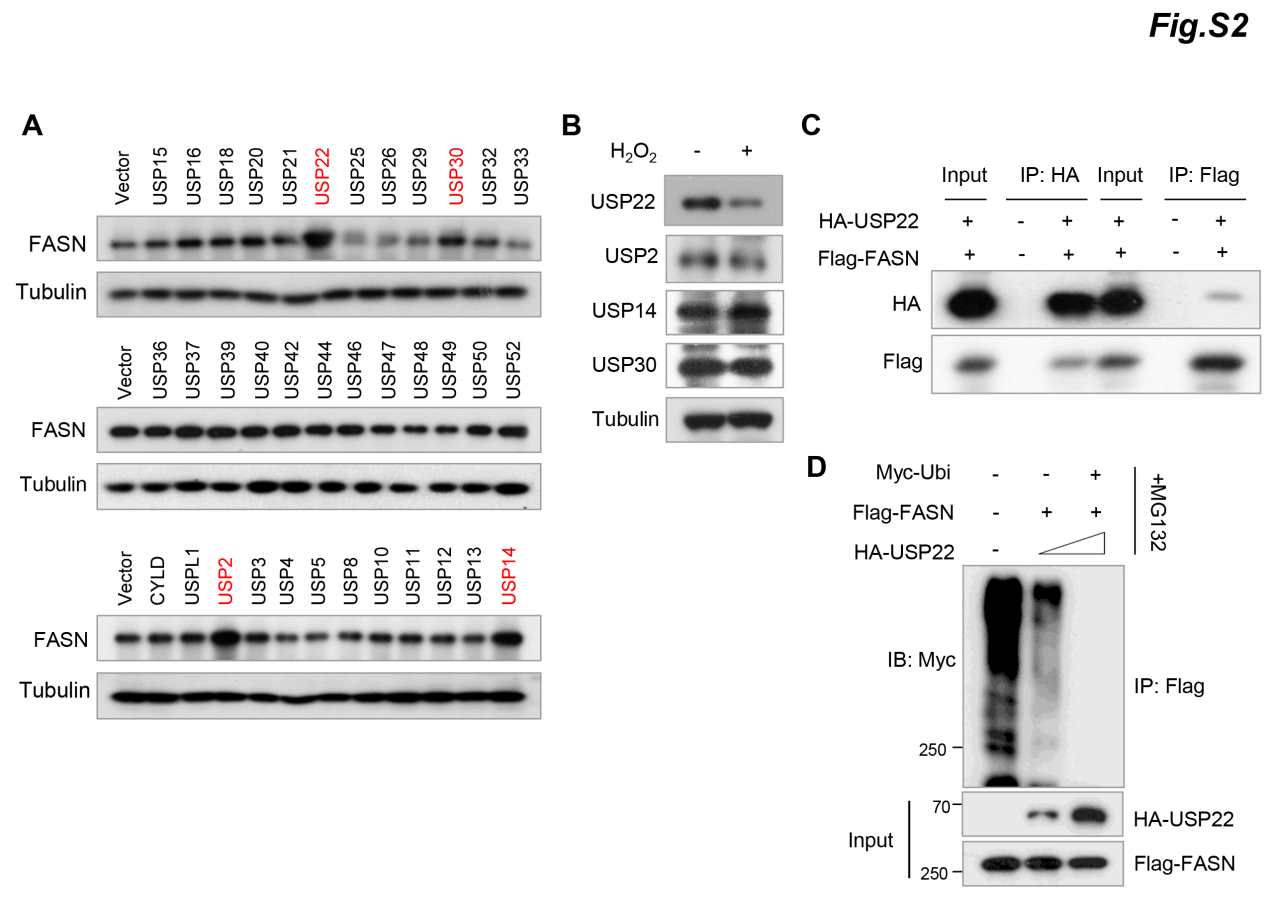
**

**Figure S2** USP22 interacts with FASN and inhibits FASN ubiquitination.

**(A)** 293T cells were transfected with the indicated deubiquitinase (DUBs), and cell lysates were analyzed by western blot using an anti-FASN antibody.

**(B)** RKO cells were treated with H_2_O_2_, and the expression of USP22, USP2, USP24, and USP30 were detected by immunoblotting.

**(C)** 293T cells were transfected with HA-USP22 and Flag-FASN, and the cell lysates were immunoprecipitated with an anti-HA or anti-Flag antibody, respectively. The resultant immunoprecipitates were subjected to western blot analysis using the indicated antibodies.

**(D)** 293T cells were transfected with Myc-ubi, Flag-FASN, and different amount of HA-USP22 plasmids. The cell lysates were immunoprecipitated with an anti-Flag antibody and then analyzed by western blotting.

**
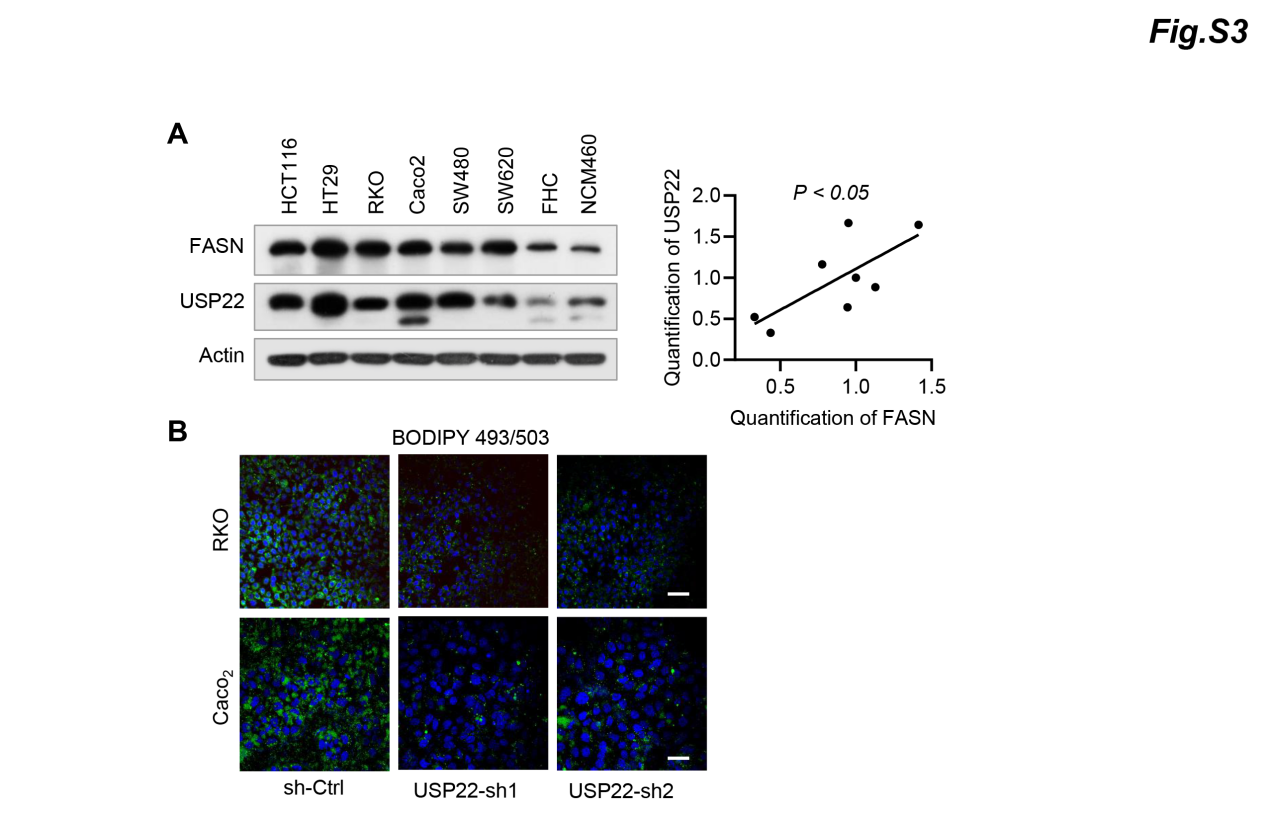
**

**Figure S3** USP22 promotes the stability of FASN.

**(A)** FASN and USP22 in different colorectal cancer cells were detected by western blotting. The FASN and USP22 expression levels in different cell lines were determined by quantification of the intensity of western blot bands, using Actin for normalization and the results are expressed as level relative to RKO cells. ^*^P<0.05.

**(B)** Cellular lipid droplets of RKO and Caco2 cells stably expressing USP22 shRNAs were visualized fluorescently with Bodipy 493/503. Representative images are shown. Scale bar, 100 µm.

**
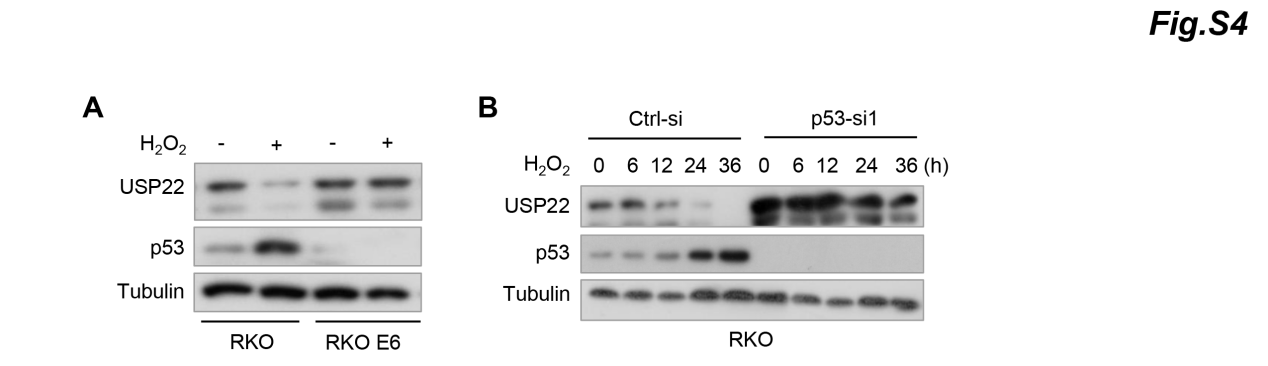
**

**Figure S4** H_2_O_2_ represses USP22 expression through p53.

**(A)** RKO and RKO E6 cells were treated with H_2_O_2_ for 24h, and cell lysates were subjected to western blot analysis using the indicated antibodies.

**(B)** RKO cells transfected with *p53* siRNA were treated with H_2_O_2_ for the indicated time intervals, and cell lysates were analyzed by immunoblotting.

**
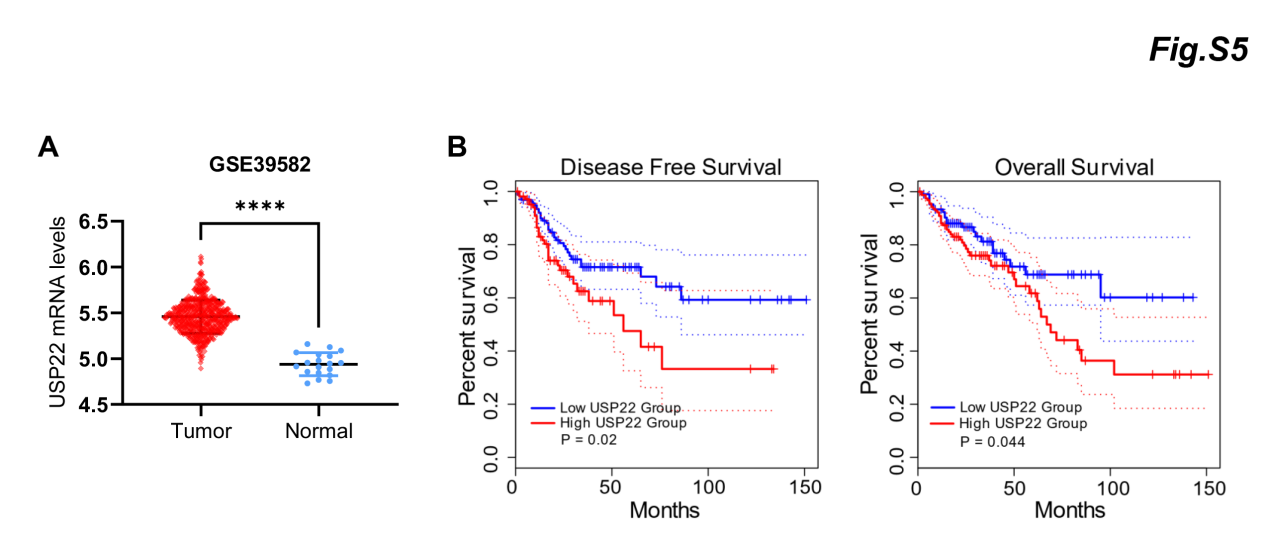
**

**Figure S5** High-level of USP22 expression predicts poor survival in colorectal cancer.

**(A)** USP22 mRNA levels between the colorectal cancer and normal tissues from GEO dataset (GSE39582).

**(B)** An analysis of TCGA datasets demonstrated that USP22 levels are correlated with colorectal cancer survival.

**Supplementary Table S1** Information of antibodies used in the study.

| **Antibody** | **Company** | **Cat. no.** | **Dilution** |
| --- | --- | --- | --- |
| USP22  USP14  USP30  USP2  Caspase3  Caspase7  Caspase9  FASN  FASN  p53  Tubulin  β-actin  Ki-67  8-OxoG  IgG  Flag  Ha  Myc  Goat anti-Mouse 488  Goat anti-Rabbit 488  Goat anti-Rabbit 594  Goat anti-Mouse 594 | ProteinTech  ProteinTech  ABclonal  ProteinTech  ProteinTech  ProteinTech  ProteinTech  ProteinTech  ProteinTech  ProteinTech  Ray Antibody  Ray Antibody  CST  Bioss  CST  Sigma  CST  CST  Invitrogen  Invitrogen  Invitrogen  Invitrogen | 55110-1-AP  14517-1-AP  A12862  10392-1-AP  66470-2-Ig  27155-1-AP  10380-1-AP  10624-2-AP  66591-1-Ig  10442-1-AP  RM2007  RM2001  9449S  Bs-1278R  2729S  F1804  3724S  2276S  A-11001  A-11034  A-11037  A-11005 | WB: 1:5000, IP: 1:500, IF/IHC, 1:200  WB: 1:2000  WB: 1:1000  WB: 1:2000  WB: 1:2000  WB: 1:2000  WB: 1:2000  WB: 1:5000, IP: 1:500, IF/IHC: 1:200  WB: 1:5000, IF/IHC: 1:200  WB: 1:2000  WB: 1:5000  WB: 1:5000  WB: 1:5000  IF/IHC: 1:200  IP: 1:500  WB: 1:2000, IP: 1:500  WB: 1:2000, IP: 1:500  WB: 1:2000, IP: 1:500  IF: 1:500  IF: 1:500  IF: 1:500  IF: 1:500 |

**Supplementary Table S2** Oligonucleotides used in the study.

| **Name** | **Sequence (5’-3’)** |
| --- | --- |
| FASN full-length CDS and deletion mutants  USP22-sh1  USP22-sh2  Primer for RT–qPCR  Human GAPDH  Human FASN  Human USP22  Primers for ChIP  p53-BS1  p53-BS2  siRNA targeting sequence  p53-siRNA1  p53-siRNA2  usp22-siRNA1  usp22-siRNA2 | CDS-F: CCGCTCGAGATGGAGGAGGTGGTGATTGCC  CDS-R: GCTCTAGACTACTTGTCATCGTCGTCCTTGTAGTC  Mut1-F: CGGAATTCatggaggaggtggtgattgcc  Mut1-R: CGGGATCCTTAgaagccaaaggagttgatgcc  Mut2-F: CGGAATTCATGggcatcaactcctttggcttc  Mut2-R: CGGGATCCTTAgaactccacaggtgggaacaa  Mut3-F: CGGAATTCATGgacaacctggagttcttcctg  Mut3-R: CGGGATCCTTAcaacaccttctgcagttctgc  Mut4-F: CGGAATTCATGgcagaactgcagaaggtgttg  Mut4-R: CGGGATCCTTAgttggtgctcatcgtctccac  Mut5-F: CGGAATTCATGgtggagacgatgagcaccaac  Mut5-R: CGGGATCCTTAggagctgtggatgatgctgat  F: CCGGAGCTACCAGGAGTCCACAAAGCTCGAGC  TTTGTGGACTCCTGGTAGCTTTTTTG  R: AATTCAAAAAAGCTACCAGGAGTCCACAAAGC  TCGAGCTTTGTGGACTCCTGGTAGCT  F: AACTCACGGACAGTCTCAACAATTTCAAGAGA  ATTGTTGAGACTGTCCGTGTTTTTC  R: TTGAGTGCCTGTCAGAGTTGTTAAAGTTCTCTT  AACAACTCTGACAGGCACAAAAAAGAGCT  F: 5’- GTCTCCTCTGACTTCAACAGCG -3’  R: 5’- ACCACCCTGTTGCTGTAGCCAA -3’  F: 5’- AAGGACCTGTCTAGGTTTGATGC -3’  R: 5’- TGGCTTCATAGGTGACTTCCA -3’  F: 5’- CCATTGATCTGATGTACGGAGG -3’  R: 5’- TCCTTGGCGATTATTTCCATGTC -3’  F：GGCTACATGCAGGTACTCGG  R：AAATGGTCTCGTTAGATTTTTCTGT  F：CAAGGGTCTGGGGGAACTTG  R：TGTGCTCGGCAATTTGTCTTG  UUAACCCUCACAAUGCACUCUGUGA  CCAUCCACUACAACUACAUGUGUAA  GCCTACCTGCTGTAAGATTAUG  GAGCTACCAGGAGTCCACAAAG |
|  | |
