# Supplementary figures and images for: Derepression of the USP22-FASN axis by p53 loss under oxidative stress drives lipogenesis and tumorigenesis
Source: Cell Death Discov. 2022 Nov 4;8:445. doi: 10.1038/s41420-022-01241-9 (PMC9636132; doi:10.1038/s41420-022-01241-9)

**Figure 1**

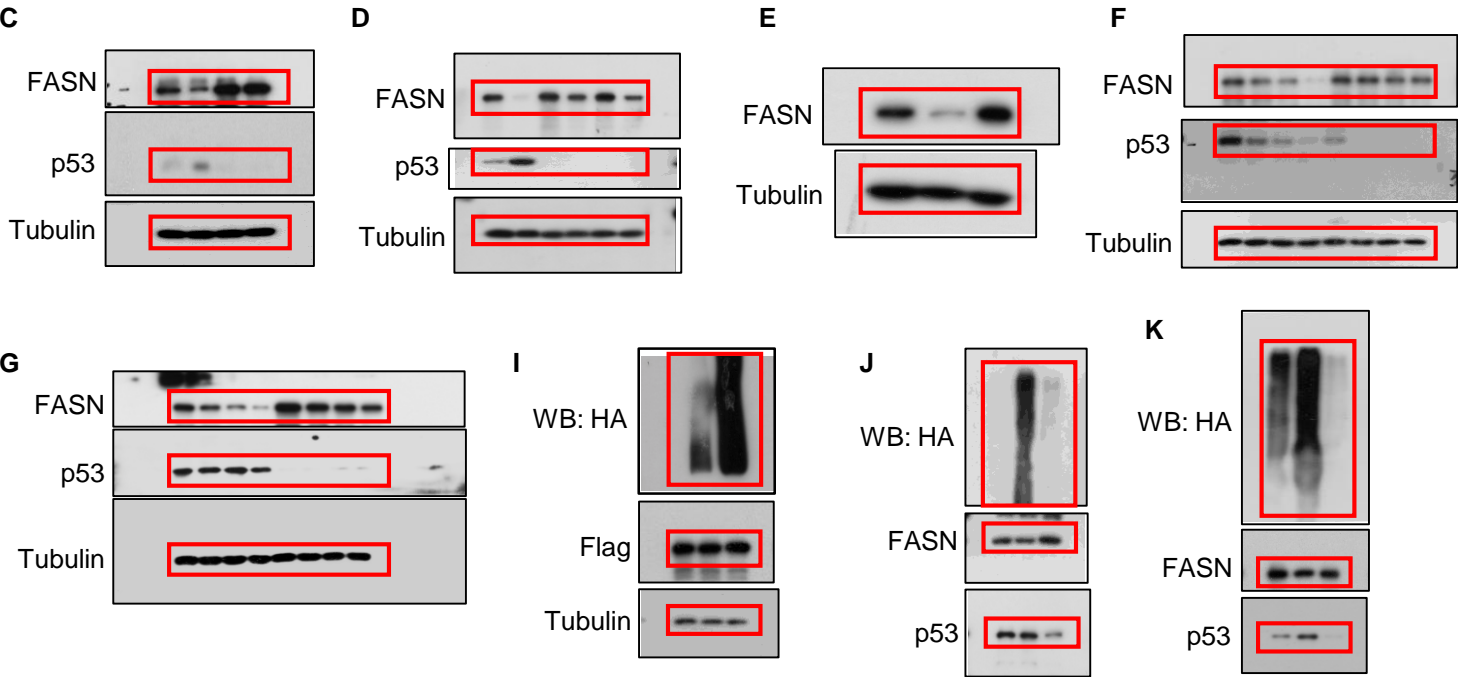

**Figure 2**

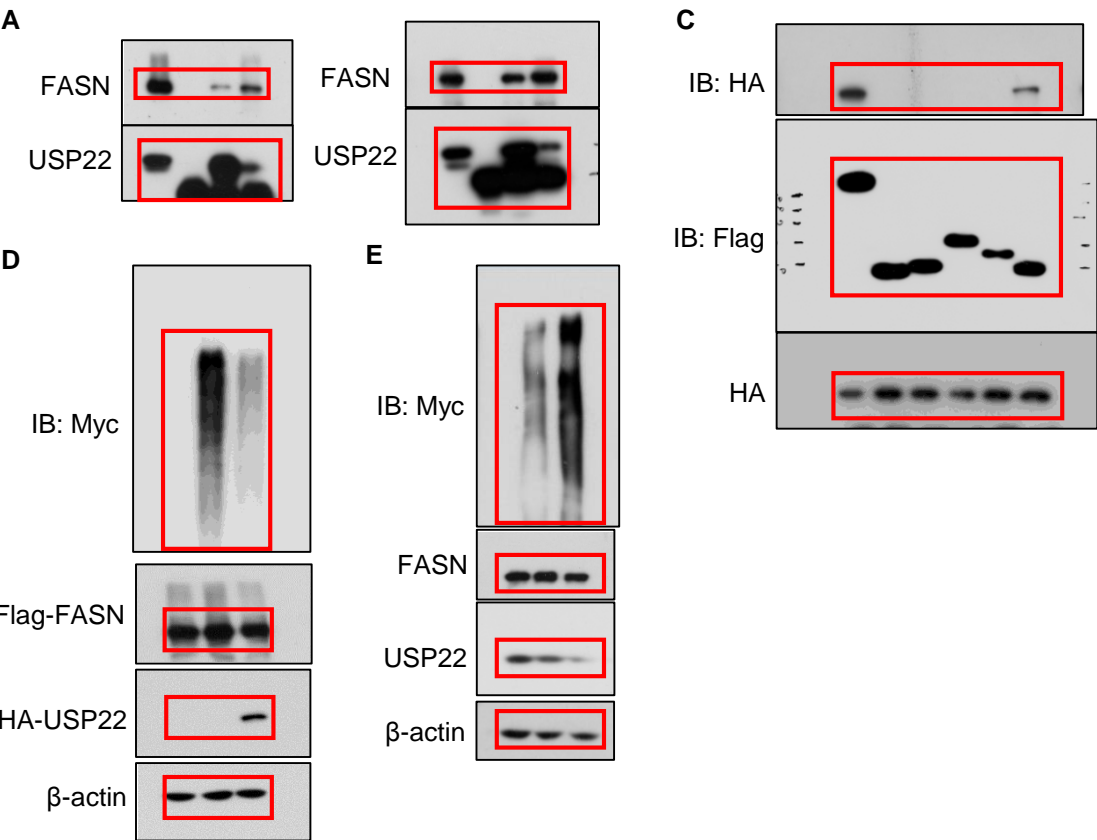

**Figure 3**

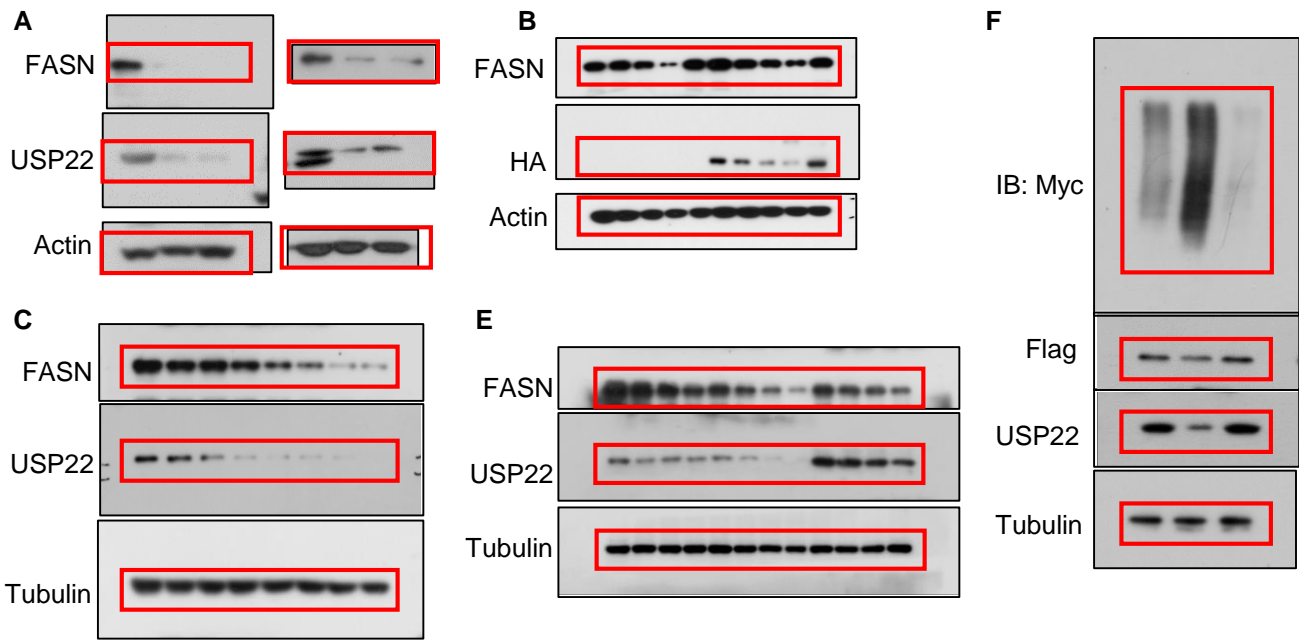

**Figure 4**

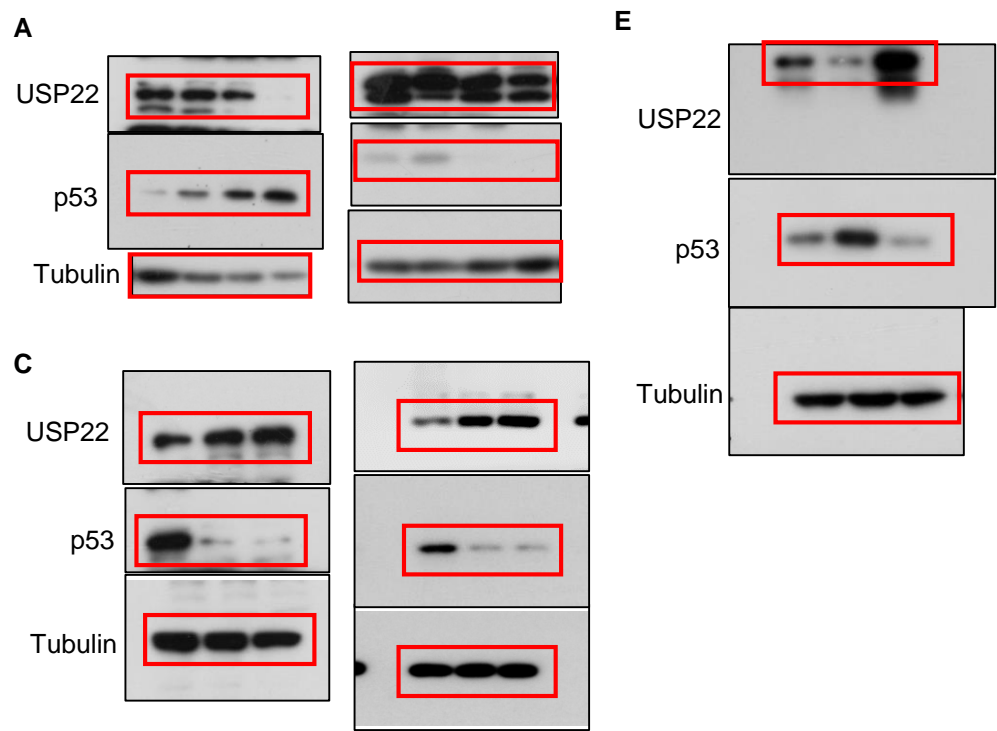

**Figure 5**

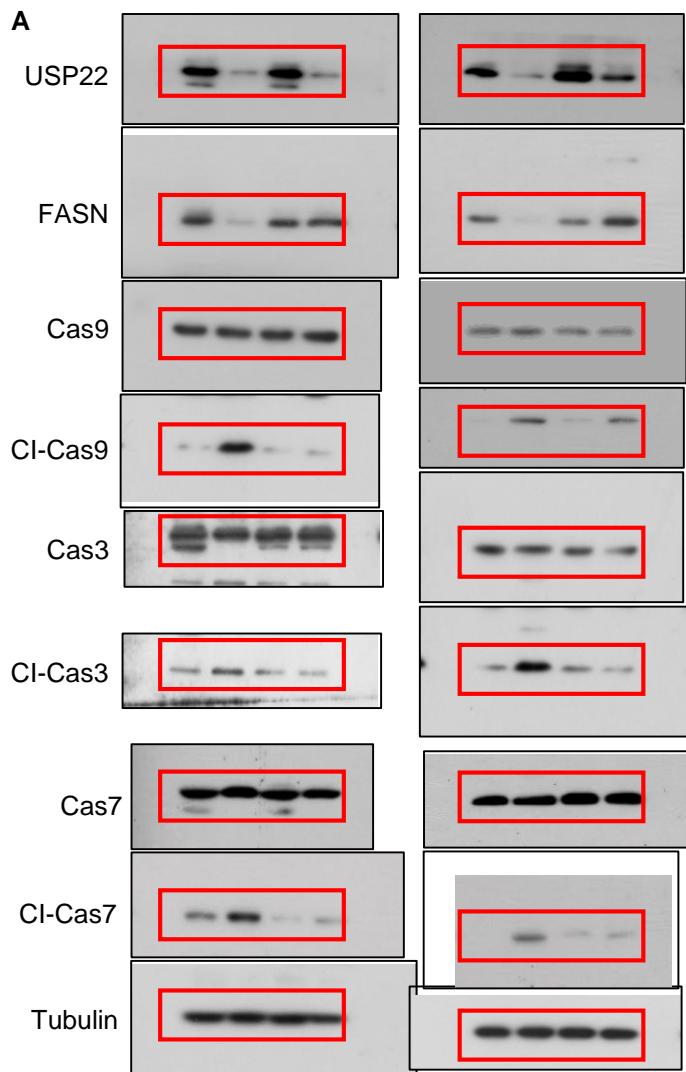

**Figure 6**

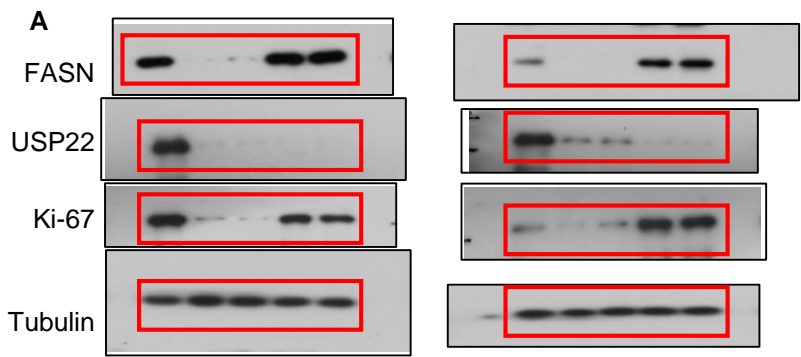

**Fig.S1**

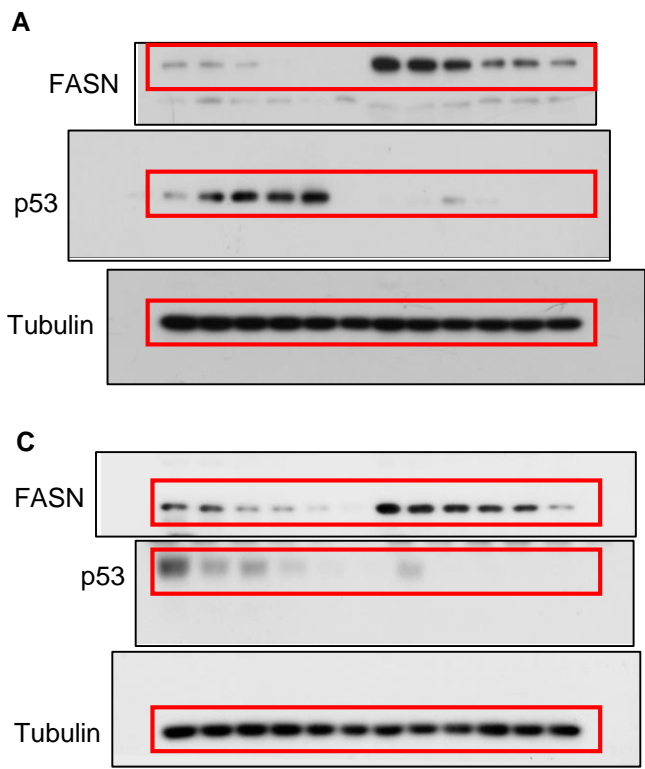

**Fig.S2**

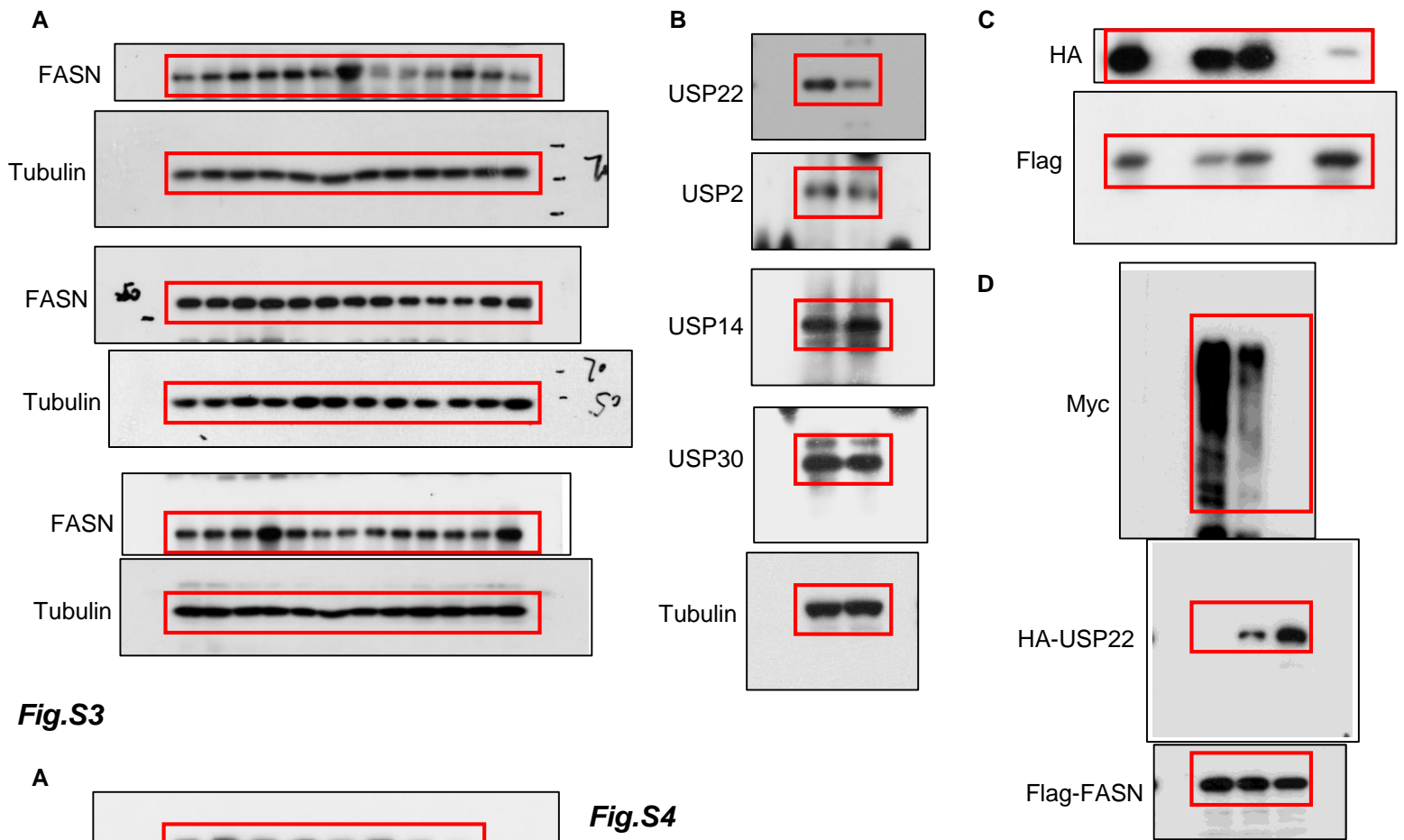

**Fig.S3**

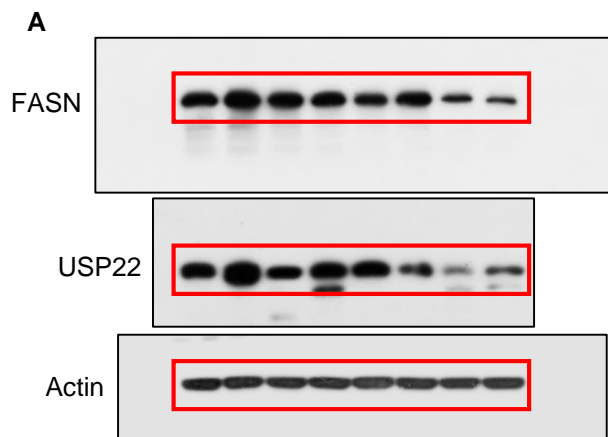

**Fig.S4**

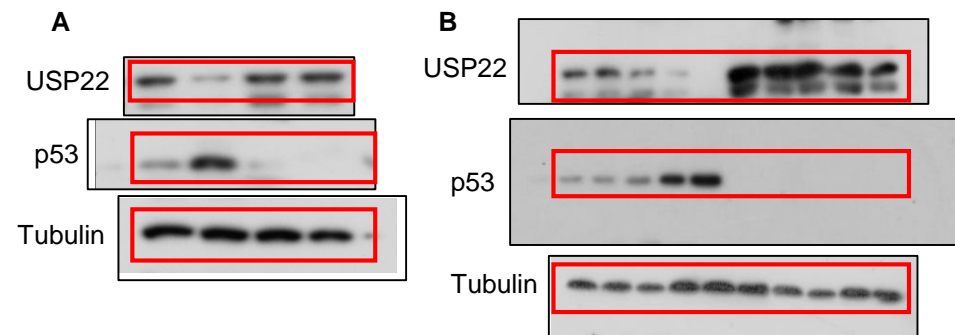

Supplement: Supplementary file 2 — Original Data File [file 41420_2022_1241_MOESM2_ESM.pdf]
