# Supplementary material for: Derepression of the USP22-FASN axis by p53 loss under oxidative stress drives lipogenesis and tumorigenesis
Source: Cell Death Discov. 2022 Nov 4;8:445. doi: 10.1038/s41420-022-01241-9 (PMC9636132; doi:10.1038/s41420-022-01241-9)
Supplement: Supplementary file 3 — Agreement of authorship changes [file 41420_2022_1241_MOESM3_ESM.pdf]

回复：Agreement of authorship changes

发起会议
2022-10-22 10:30:47

发件人： "韩泽龙" <hzl198886@163.com>

收件人： "周爱冬" <aidern0927@smu.edu.cn>

Yes

---- 回复的原邮件 ----

发件人 周爱冬<aidern0927@smu. edu. cn>

日期 2022年10月22日 10:28

收件人 hzl198886@163. com<hzl198886@163. com>、ming. liu@kuleuven. be<ming. liu@kuleuven. be>、1085594190@qq. com<1085594190@qq. com>、1140234753@qq. com<1140234753@qq. com>、1484968667@qq. com<1484968667@qq. com>、1444233436@qq. com<1444233436@qq. com>、wli817@126. com<wli817@126. com>、13533758584@163. com<13533758584@163. com>、3104761566@qq. com<3104761566@qq. com>、gzzengyu@163. com<gzzengyu@163. com>、zhan781135915@163. com<zhan781135915@163. com>、20628315@qq. com<20628315@qq. com>、gczhkq@163. com<gczhkq@163. com>、zhuxx01@126. com<zhuxx01@126. com>、liuside2011@163. com<liuside2011@163. com>、luoxiaobei63@126. com<luoxiaobei63@126. com>

主题 Agreement of authorship changes

Dear collaborators,

Our manuscript (CDDISCOVERY-22-5061R1) has now been principally accepted by Cell Death Discovery. We added Yuxin Xie as co-first author, and Ziling zhan and Yingzhuo Lin as co-authors because of their contributions during the revision of the paper. If you agree with the changes of authorship, please reply yes to this email directly.

Thank you for your cooperation!

Sincerely,

Aidong

回复：Agreement of authorship changes

发起会议
2022-10-22 10:34:41

发件人： "gczhkq" <gczhkq@163.com>

收件人： "周爱冬" <aidern0927@smu.edu.cn>

抄 送： "hzl198886" <hzl198886@163.com> "ming.liu" <ming.liu@kuleuven.be> "1085594190" <1085594190@qq.com> "1140234753" <1140234753@qq.com> "1484968667" <1484968667@qq.com> .. [↓还有10个联系人]

Yes

---- 回复的原邮件 ----

发件人	周爱冬<aidern0927@smu.edu.cn>
日期	2022年10月22日 10:28
收件人	hzl198886@163.com<hzl198886@163.com>、ming.liu@kuleuven.be<ming.liu@kuleuven.be>、1085594190@qq.com<1085594190@qq.com>、1140234753@qq.com<1140234753@qq.com>、1484968667@qq.com<1484968667@qq.com>、1444233436@qq.com<1444233436@qq.com>、wli817@126.com<wli817@126.com>、13533758584@163.com<13533758584@163.com>、3104761566@qq.com<3104761566@qq.com>、gzzengyu@163.com<gzzengyu@163.com>、zhan781135915@163.com<zhan781135915@163.com>、20628315@qq.com<20628315@qq.com>、gczhkq@163.com<gczhkq@163.com>、zhuxx01@126.com<zhuxx01@126.com>、liuside2011@163.com<liuside2011@163.com>、luoxiaobei63@126.com<luoxiaobei63@126.com>
主题	Agreement of authorship changes

Dear collaborators,

Our manuscript (CDDISCOVERY-22-5061R1) has now been principally accepted by Cell Death Discovery. We added Yuxin Xie as co-first author, and Ziling zhan and Yingzhuo Lin as co-authors because of their contributions during the revision of the paper. If you agree with the changes of authorship, please reply yes to this email directly.

Thank you for your cooperation!

Sincerely,

回复 | 回复全部 | 转发 | 移动到 | 标记为 | 更多 | 删除

Re: Agreement of authorship changes

发起会议
2022-10-22 10:36:57

发件人： "Lycoris Radiata" <3104761566@qq.com>

收件人： "周爱冬" <aidern0927@smu.edu.cn>

yes

---Original---

From: "周爱冬"<aidern0927@smu.edu.cn>
Date: Sat, Oct 22, 2022 10:28 AM
To: "hzl198886"<hzl198886@163.com>;"ming.liu"<ming.liu@kuleuven.be>;"1085594190"<1085594190@qq.com>;"1140234753"<1140234753@qq.com>;"1484968667"<1484968667@qq.com>;"1444233436"<1444233436@qq.com>;"wli817"<wli817@126.com>;"13533758584"<13533758584@163.com>;"3104761566"<3104761566@qq.com>;"gzzengyu"<gzzengyu@163.com>;"zhan781135915"<zhan781135915@163.com>;"20628315"<20628315@qq.com>;"gczhkq"<gczhkq@163.com>;"zhuxx01"<zhuxx01@126.com>;"liuside2011"<liuside2011@163.com>;"luoxiaobei63"<luoxiaobei63@126.com>;
Subject: Agreement of authorship changes

Dear collaborators,

Our manuscript (CDDISCOVERY-22-5061R1) has now been principally accepted by Cell Death Discovery. We added Yuxin Xie as co-first author, and Ziling zhan and Yingzhuo Lin as co-authors because of their contributions during the revision of the paper. If you agree with the changes of authorship, please reply yes to this email directly.

Thank you for your cooperation!

Sincerely,

Aidong

Aidong Zhou, PhD

Professor and Associate Director

Department of Cell Biology, School of Basic Medical Sciences

快捷回复给所有人

回复：Agreement of authorship changes

2022-10-22 10:40:31

发件人： "Xie Yuxin" <1085594190@qq.com>

收件人： "周爱冬" <aidern0927@smu.edu.cn>

发起会议

Yes!

------------------ 原始邮件 ------------------

发件人: "周爱冬" <aidern0927@smu.edu.cn>;
发送时间: 2022年10月22日(星期六) 上午10:28
收件人: "hzl198886"<hzl198886@163.com>;"ming.liu"<ming.liu@kuleuven.be>;"Xie Yuxin"<1085594190@qq.com>;"1140234753"<1140234753@qq.com>;"1484968667"<1484968667@qq.com>;"1444233436"<1444233436@qq.com>;"wli817"<wli817@126.com>;"13533758584"<13533758584@163.com>;"3104761566"<3104761566@qq.com>;"gzzengyu"<gzzengyu@163.com>;"zhan781135915"<zhan781135915@163.com>;"20628315"<20628315@qq.com>;"gczhkq"<gczhkq@163.com>;"zhuxx01"<zhuxx01@126.com>;"liuside2011"<liuside2011@163.com>;"luoxiaobei63"<luoxiaobei63@126.com>;
主题: Agreement of authorship changes

Dear collaborators,

Our manuscript (CDDISCOVERY-22-5061R1) has now been principally accepted by Cell Death Discovery. We added Yuxin Xie as co-first author, and Ziling zhan and Yingzhuo Lin as co-authors because of their contributions during the revision of the paper. If you agree with the changes of authorship, please reply yes to this email directly.

Thank you for your cooperation!

Sincerely,

Aidong

Aidong Zhou, PhD

快捷回复给所有人

Re: Agreement of authorship changes

2022-10-22 10:46:51

发件人： "罗晓蓓" <luoxiaobei63@126.com>

收件人： "周爱冬" <aidern0927@smu.edu.cn>

抄 送： "韩泽龙" <hzl198886@163.com> "ming.liu" <ming.liu@kuleuven.be> "1085594190" <1085594190@qq.com> "1140234753" <1140234753@qq.com> "1484968667" <1484968667@qq.com> .. [↓还有10个联系人]

Dear Prof Zhou,

Yes, I agree with the authorship changes.

Thank you for your kind notifications.

Best,
Xiaobei

---- Replied Message ----

From	周爱冬<aidern0927@smu.edu.cn>
Date	10/22/2022 10:28
To	hzl198886<hzl198886@163.com>,<br>ming.liu<ming.liu@kuleuven.be>,<br>1085594190<1085594190@qq.com>,<br>1140234753<1140234753@qq.com>,<br>1484968667<1484968667@qq.com>,<br>1444233436<1444233436@qq.com>,<br>wli817<wli817@126.com>,<br>13533758584<13533758584@163.com>,<br>3104761566<3104761566@qq.com>,<br>gzzengyu<gzzengyu@163.com>,<br>zhan781135915<zhan781135915@163.com>,<br>20628315<20628315@qq.com>,<br>gczhkq<gczhkq@163.com>,<br>zhuxx01<zhuxx01@126.com>,<br>liuside2011<liuside2011@163.com>,<br>luoxiaobei63<luoxiaobei63@126.com>
Subject	Agreement of authorship changes

Dear collaborators,

回复：Agreement of authorship changes

发起会议
2022-10-22 17:11:22

发件人："小卓子" <20628315@qq.com>
收件人："周爱冬" <aidern0927@smu.edu.cn>

yes

------------------ 原始邮件 ------------------

发件人: "周爱冬" <aidern0927@smu.edu.cn>;
发送时间: 2022年10月22日(星期六) 上午10:28
收件人: "hzl198886"<hzl198886@163.com>;"ming.liu"<ming.liu@kuleuven.be>;"1085594190"<1085594190@qq.com>;"1140234753"<1140234753@qq.com>;"1484968667"<1484968667@qq.com>;"1444233436"<1444233436@qq.com>;"wli817"<wli817@126.com>;"13533758584"<13533758584@163.com>;"3104761566"<3104761566@qq.com>;"gzzengyu"<gzzengyu@163.com>;"zhan781135915"<zhan781135915@163.com>;"小卓子"<20628315@qq.com>;"gczhkq"<gczhkq@163.com>;"zhuxx01"<zhuxx01@126.com>;"liuside2011"<liuside2011@163.com>;"luoxiaobei63"<luoxiaobei63@126.com>;
主题: Agreement of authorship changes

Dear collaborators,

Our manuscript (CDDISCOVERY-22-5061R1) has now been principally accepted by Cell Death Discovery. We added Yuxin Xie as co-first author, and Ziling zhan and Yingzhuo Lin as co-authors because of their contributions during the revision of the paper. If you agree with the changes of authorship, please reply yes to this email directly.

Thank you for your cooperation!

Sincerely,

Aidong

Aidong Zhou, PhD

Re: Agreement of authorship changes

发起会议

2022-10-22 14:06:20

发件人： "Ming Liu" <ming.liu@kuleuven.be>

收件人： "周爱冬" <aidern0927@smu.edu.cn>

抄 送： "hzl198886@163.com" <hzl198886@163.com> "1085594190@qq.com" <1085594190@qq.com> "1140234753@qq.com" <1140234753@qq.com> "1484968667@qq.com" <1484968667@qq.com> "1444233436@qq.com" <1444233436@qq.com> .. [↓还有10个联系人]

Dear Prof. Zhou,

Yes.

Best regards,
Ming

On Oct 22, 2022, at 04:36, 周爱冬 <aidern0927@smu.edu.cn> wrote:

Dear collaborators,

Our manuscript (CDDISCOVERY-22-5061R1) has now been principally accepted by Cell Death Discovery. We added Yuxin Xie as co-first author, and Ziling zhan and Yingzhuo Lin as co-authors because of their contributions during the revision of the paper. If you agree with the changes of authorship, please reply yes to this email directly.

Thank you for your cooperation!

Sincerely,

Aidong

Re:Agreement of authorship changes

发起会议

2022-10-22 21:03:32

发件人 : "ZhanHC" <zhan781135915@163.com>

收件人 : "周爱冬" <aidern0927@smu.edu.cn>

Dear Prof Zhou,

Yes, I agree.

Best,
Hongchao

At 2022-10-22 10:28:42, "周爱冬" <aidern0927@smu.edu.cn> wrote:

> Dear collaborators,
>
> Our manuscript (CDDISCOVERY-22-5061R1) has now been principally accepted by Cell Death Discovery. We added Yuxin Xie as co-first author, and Ziling zhan and Yingzhuo Lin as co-authors because of their contributions during the revision of the paper. If you agree with the changes of authorship, please reply yes to this email directly.
>
> Thank you for your cooperation!
>
> Sincerely,

回复 回复全部 转发 移动到 标记为 更多 删除

Re:Agreement of authorship changes

发件人："Xiaoxia Chen" <m13533758584@163.com>

收件人："周爱冬" <aidern0927@smu.edu.cn>

抄 送：hzl198886@163.com ming.liu@kuleuven.be 1085594190@qq.com 1140234753@qq.com 1484968667@qq.com

.. [↓还有10个联系人]

发起会议
2022-10-22 21:04:45

yes

在 2022-10-22 10:28:42，"周爱冬" <aidern0927@smu.edu.cn> 写道：

> Dear collaborators,
>
> Our manuscript (CDDISCOVERY-22-5061R1) has now been principally accepted by Cell Death Discovery. We added Yuxin Xie as co-first author, and Ziling zhan and Yingzhuo Lin as co-authors because of their contributions during the revision of the paper. If you agree with the changes of authorship, please reply yes to this email directly.
>
> Thank you for your cooperation!
>
> Sincerely,
>
> Aidong

Aidong Zhou, PhD

Professor and Associate Director
Department of Cell Biology, School of Basic Medical Sciences
Southern Medical University
Guangzhou, China
Tel: (020)6164 8214
Email: aidern0927@smu.edu.cn

快捷回复给所有人

回复 回复全部 转发 移动到 标记为 更多 删除

回复：Agreement of authorship changes

发起会议
2022-10-22 21:51:06

发件人： " ↘"Mr,林" <1140234753@qq.com>

收件人： "周爱冬" <aidern0927@smu.edu.cn> "hzl198886" <hzl198886@163.com> "ming.liu" <ming.liu@kuleuven.be> "1085594190" <1085594190@qq.com> "1484968667" <1484968667@qq.com> .. [↓还有11个联系人]

yes

------------------ 原始邮件 ------------------

发件人： "周爱冬"<aidern0927@smu.edu.cn>;
发送时间： 2022年10月22日(星期六) 上午10:28
收件人： "hzl198886"<hzl198886@163.com>; "ming.liu"<ming.liu@kuleuven.be>; "1085594190"<1085594190@qq.com>; " ↘''Mr,林" <1140234753@qq.com>; "1484968667"<1484968667@qq.com>; "1444233436"<1444233436@qq.com>; "wli817"<wli817@126.com>; "13533758584" <13533758584@163.com>; "3104761566"<3104761566@qq.com>; "gzzengyu"<gzzengyu@163.com>; "zhan781135915"<zhan781135915@163.com>; "20628315"<20628315@qq.com>; "gczhkq"<gczhkq@163.com>; "zhuxx01"<zhuxx01@126.com>; "liuside2011"<liuside2011@163.com>; "luoxiaobei63" <luoxiaobei63@126.com>;
主题： Agreement of authorship changes

Dear collaborators,

Our manuscript (CDDISCOVERY-22-5061R1) has now been principally accepted by Cell Death Discovery. We added Yuxin Xie as co-first author, and Ziling zhan and Yingzhuo Lin as co-authors because of their contributions during the revision of the paper. If you agree with the changes of authorship, please reply yes to this email directly.

Thank you for your cooperation!

Sincerely,

Aidong

Aidong Zhou, PhD

回复 回复全部 转发 移动到 标记为 更多 删除

回复：Agreement of authorship changes

发起会议
2022-10-22 21:52:22

发件人："ヾ"Mr,林" <1140234753@qq.com>

收件人："周爱冬" <aidern0927@smu.edu.cn>

yes

------------------ 原始邮件 ------------------

发件人: "周爱冬" <aidern0927@smu.edu.cn>;
发送时间: 2022年10月22日(星期六) 上午10:28
收件人: "hzl198886"<hzl198886@163.com>;"ming.liu"<ming.liu@kuleuven.be>;"1085594190"<1085594190@qq.com>;"ヾ''Mr,林"<1140234753@qq.com>;"1484968667"<1484968667@qq.com>;"1444233436"<1444233436@qq.com>;"wli817"<wli817@126.com>;"13533758584"<13533758584@163.com>;"3104761566"<3104761566@qq.com>;"gzzengyu"<gzzengyu@163.com>;"zhan781135915"<zhan781135915@163.com>;"20628315"<20628315@qq.com>;"gczhkq"<gczhkq@163.com>;"zhuxx01"<zhuxx01@126.com>;"liuside2011"<liuside2011@163.com>;"luoxiaobei63"<luoxiaobei63@126.com>;
主题: Agreement of authorship changes

Dear collaborators,

Our manuscript (CDDISCOVERY-22-5061R1) has now been principally accepted by Cell Death Discovery. We added Yuxin Xie as co-first author, and Ziling zhan and Yingzhuo Lin as co-authors because of their contributions during the revision of the paper. If you agree with the changes of authorship, please reply yes to this email directly.

Thank you for your cooperation!

Sincerely,

Aidong

Aidong Zhou, PhD

快捷回复给所有人

回复 | 回复全部 | 转发 | 移动到 | 标记为 | 更多 | 删除

Re:Agreement of authorship changes

发起会议
2022-10-22 23:29:05

发件人： "刘思德" <liuside2011@163.com>

收件人： "周爱冬" <aidern0927@smu.edu.cn>

抄 送： hzl198886@163.com ming.liu@kuleuven.be 1085594190@qq.com 1140234753@qq.com 1484968667@qq.com
.. [↓还有10个联系人]

Yes

At 2022-10-22 10:28:42, "周爱冬" <aidern0927@smu.edu.cn> wrote:

> Dear collaborators,
>
> Our manuscript (CDDISCOVERY-22-5061R1) has now been principally accepted by Cell Death Discovery. We added Yuxin Xie as co-first author, and Ziling zhan and Yingzhuo Lin as co-authors because of their contributions during the revision of the paper. If you agree with the changes of authorship, please reply yes to this email directly.
>
> Thank you for your cooperation!
>
> Sincerely,
>
> ...

回复 回复全部 转发 移动到 标记为 更多 删除

Re: Agreement of authorship changes

发起会议
2022-10-23 07:09:08

发件人： "Sam" <gzzengyu@163.com>

收件人： "周爱冬" <aidern0927@smu.edu.cn>

Yes.

Sam
邮箱：gzzengyu@163.com

---- Replied Message ----

From	周爱冬<aidern0927@smu.edu.cn>
Date	10/22/2022 10:28
To	hzl198886<hzl198886@163.com>, ming.liu<ming.liu@kuleuven.be>, 1085594190<1085594190@qq.com>, 1140234753<1140234753@qq.com>, 1484968667<1484968667@qq.com>, 1444233436<1444233436@qq.com>, wli817<wli817@126.com>, 13533758584<13533758584@163.com>, 3104761566<3104761566@qq.com>, gzzengyu<gzzengyu@163.com>, zhan781135915<zhan781135915@163.com>, 20628315<20628315@qq.com>, gczhkq<gczhkq@163.com>, zhuxx01<zhuxx01@126.com>, liuside2011<liuside2011@163.com>, luoxiaobei63<luoxiaobei63@126.com>
Subject	Agreement of authorship changes

Dear collaborators,

Our manuscript (CDDISCOVERY-22-5061R1) has now been principally accepted by Cell Death Discovery. We added Yuxin Xie as co-first author, and Ziling zhan and Yingzhuo Lin as co-authors because of their contributions during the revision of the paper. If you agree with the changes of authorship, please reply yes to this email directly.

回复 | 回复全部 | 转发 | 移动到 | 标记为 | 更多 | 删除

回复：Agreement of authorship changes

发起会议
2022-10-23 10:22:44

发件人： "邮件126管理员" <zhuxx01@126.com>

收件人： "周爱冬" <aidern0927@smu.edu.cn>

Yes

zhuxx01@126.com

邮箱：zhuxx01@126.com

---- 回复的原邮件 ----

发件人	周爱冬<aidern0927@smu.edu.cn>
日期	2022年10月22日 10:28
收件人	hzl198886@163.com<hzl198886@163.com>、ming.liu@kuleuven.be<ming.liu@kuleuven.be>、1085594190@qq.com<1085594190@qq.com>、1140234753@qq.com<1140234753@qq.com>、1484968667@qq.com<1484968667@qq.com>、1444233436@qq.com<1444233436@qq.com>、wli817@126.com<wli817@126.com>、13533758584@163.com<13533758584@163.com>、3104761566@qq.com<3104761566@qq.com>、gzzengyu@163.com<gzzengyu@163.com>、zhan781135915@163.com<zhan781135915@163.com>、20628315@qq.com<20628315@qq.com>、gczhkq@163.com<gczhkq@163.com>、zhuxx01@126.com<zhuxx01@126.com>、liuside2011@163.com<liuside2011@163.com>、luoxiaobei63@126.com<luoxiaobei63@126.com>
主题	Agreement of authorship changes

Dear collaborators,

Our manuscript (CDDISCOVERY-22-5061R1) has now been principally accepted by Cell Death Discovery. We added Yuxin Xie as co-first author, and Ziling zhan and Yingzhuo Lin as co-authors because of their contributions during the revision of the paper. If you agree with the changes of authorship, please reply yes to this email directly.

Thank you for your cooperation!

回复 回复全部 转发 移动到 标记为 更多 删除

回复：Agreement of authorship changes

发起会议
2022-10-22 21:13:20

发件人： "zilingzhan" <1484968667@qq.com>

收件人： "周爱冬" <aidern0927@smu.edu.cn>

Dear Prof. Zhou,

Yes.

Best regards,

Ziling

------------------ 原始邮件 ------------------

发件人： "周爱冬" <aidern0927@smu.edu.cn>;
发送时间： 2022年10月22日(星期六) 上午10:28
收件人： "hzl198886"<hzl198886@163.com>;"ming.liu"<ming.liu@kuleuven.be>;"1085594190"<1085594190@qq.com>;"1140234753"<1140234753@qq.com>;"zilingzhan"<1484968667@qq.com>;"1444233436"<1444233436@qq.com>;"wli817"<wli817@126.com>;"13533758584"<13533758584@163.com>;"3104761566"<3104761566@qq.com>;"gzzengyu"<gzzengyu@163.com>;"zhan781135915"<zhan781135915@163.com>;"20628315"<20628315@qq.com>;"gczhkq"<gczhkq@163.com>;"zhuxx01"<zhuxx01@126.com>;"liuside2011"<liuside2011@163.com>;"luoxiaobei63"<luoxiaobei63@126.com>;
主题： Agreement of authorship changes

Dear collaborators,

Our manuscript (CDDISCOVERY-22-5061R1) has now been principally accepted by Cell Death Discovery. We added Yuxin Xie as co-first author, and Ziling zhan and Yingzhuo Lin as co-authors because of their contributions during the revision of the paper. If you agree with the changes of authorship, please reply yes to this email directly.

Thank you for your cooperation!

Sincerely,

回复 | 回复全部 | 转发 | 移动到 | 标记为 | 更多 | 删除

Re: Agreement of authorship changes

wangli 发送给 周爱冬

发起会议

2022-10-23 10:27:32

yes

发自 网易邮箱大师

---- 回复的原邮件 ----

发件人	周爱冬<aidern0927@smu.edu.cn>
日期	2022年10月22日 10:28
收件人	hzl198886@163.com<hzl198886@163.com>、ming.liu@kuleuven.be<ming.liu@kuleuven.be>、1085594190@qq.com<1085594190@qq.com>、1140234753@qq.com<1140234753@qq.com>、1484968667@qq.com<1484968667@qq.com>、1444233436@qq.com<1444233436@qq.com>、wli817@126.com<wli817@126.com>、13533758584@163.com<13533758584@163.com>、3104761566@qq.com<3104761566@qq.com>、gzzengyu@163.com<gzzengyu@163.com>、zhan781135915@163.com<zhan781135915@163.com>、20628315@qq.com<20628315@qq.com>、gczhkq@163.com<gczhkq@163.com>、zhuxx01@126.com<zhuxx01@126.com>、liuside2011@163.com<liuside2011@163.com>、luoxiaobei63@126.com<luoxiaobei63@126.com>
主题	Agreement of authorship changes

Dear collaborators,

Our manuscript (CDDISCOVERY-22-5061R1) has now been principally accepted by Cell Death Discovery. We added Yuxin Xie as co-first author, and Ziling zhan and Yingzhuo Lin as co-authors because of their contributions during the revision of the paper. If you agree with the changes of authorship, please reply yes to this email directly.

Thank you for your cooperation!

Sincerely,

Aidong
